# Distinguishing bulk redox from near-surface degradation in lithium nickel oxide cathodes†

**DOI:** 10.1039/d4ee02398f

**Published:** 2024-09-13

**Authors:** Lijin An, Jack E. N. Swallow, Peixi Cong, Ruomu Zhang, Andrey D. Poletayev, Erik Björklund, Pravin N. Didwal, Michael W. Fraser, Leanne A. H. Jones, Conor M. E. Phelan, Namrata Ramesh, Grant Harris, Christoph J. Sahle, Pilar Ferrer, David C. Grinter, Peter Bencok, Shusaku Hayama, M. Saiful Islam, Robert House, Peter D. Nellist, Robert J. Green, Rebecca J. Nicholls, Robert S. Weatherup

**Affiliations:** a Department of Materials, University of Oxford Parks Road Oxford OX1 3PH UK robert.weatherup@materials.ox.ac.uk; b The Faraday Institution, Quad One, Harwell Science and Innovation Campus Didcot OX11 0RA UK; c Department of Physics and Engineering Physics, University of Saskatchewan Saskatoon Canada S7N 5E2; d ESRF – The European Synchrotron 71 Avenue des Martyrs 38000 Grenoble France; e Diamond Light Source, Harwell Science and Innovation Campus Didcot OX11 0DE UK; f Stewart Blusson Quantum Matter Institute, University of British Columbia Vancouver Canada V6T 1Z1

## Abstract

Ni-rich layered oxide cathodes can deliver higher energy density batteries, but uncertainties remain over their charge compensation mechanisms and the degradation processes that limit cycle life. Trapped molecular O_2_ has been identified within LiNiO_2_ at high states of charge, as seen for Li-rich cathodes where excess capacity is associated with reversible oxygen redox. Here we show that bulk redox in LiNiO_2_ occurs by Ni–O rehybridization, lowering the electron density on O sites, but importantly without the involvement of molecular O_2_. Instead, trapped O_2_ is related to degradation at surfaces in contact with the electrolyte, and is accompanied by Ni reduction. O_2_ is removed on discharge, but excess Ni^2+^ persists forming a reduced surface layer, associated with impeded Li transport. This implicates the instability of delithiated LiNiO_2_ in contact with the electrolyte in surface degradation through O_2_ formation and Ni reduction, highlighting the importance of surface stabilisation strategies in suppressing LiNiO_2_ degradation.

## Abstract 2

Bulk redox activity in LiNiO_2_ proceeds without significant involvement of molecular oxygen, whose formation is instead associated with surface degradation.

## Graphical abstract

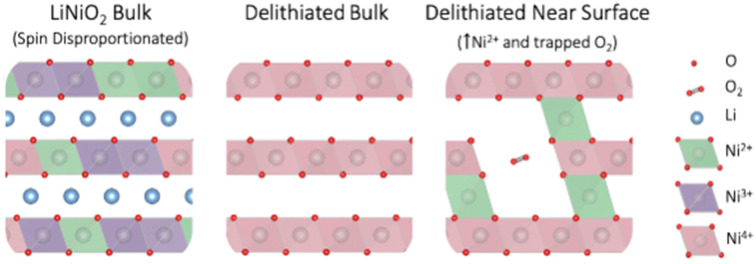

**Broader context**

Increasing the capacity of Li-ion batteries requires cathodes which can reversibly deintercalate more lithium without leading to structural instability and severe capacity fade. To this end, Ni-rich layered cathodes are under development for next-generation batteries, with LiNiO_2_ the archetypal system for investigating their charging mechanisms. However, the role played by different redox centres in LiNiO_2_ is still debated, and the connections with structural instabilities and associated degradation are not yet fully established. Recent reports have suggested the involvement of molecular O_2_ in the bulk redox process at high states of charge, with direct experimental detection of O_2_ based on techniques that probe 100–200 nm into the surface of the few μm-sized cathode particles. Here, we combine a broad suite of X-ray spectroscopies with varying information depths (10 nm to 10 μm) to separate the bulk redox from surface degradation. We reveal that trapped O_2_ formation in LiNiO_2_ is primarily associated with degradation at surfaces in contact with the electrolyte, rather than contributing to the bulk redox process. Interpretation of experimental spectra using theoretical calculations shows that bulk charge compensation proceeds by Ni–O rehybridization. These findings highlight the importance of using bulk sensitive techniques to understand redox, and suggests design strategies for stabilising high energy density Ni-rich cathodes.

## Introduction

Layered transition metal (TM) oxides, LiTMO_2_ (TM = Co, Ni, Mn, *etc.*), are the cathode materials of choice for commercial high-energy density Li-ion batteries, reversibly intercalating Li over thousands of cycles.^1,2^ Ni-rich stoichiometries are increasingly favoured to increase capacity, and lower Co content, which is expensive and has ethical concerns around its mining.^3^ In the traditional picture of charge compensation, Li^+^ removal is compensated by an increase in the formal oxidation state of the redox-active TM centres *via* a single electron transfer. However, it is well-established that there are accompanying changes in TM–O bond covalency meaning both TM and O sites are involved.^4–9^

The archetypal Ni-rich cathode material, LiNiO_2_, undergoes several first-order structural phase transitions upon delithiation, with significant degradation observed at high potentials that has been associated with severe lattice changes,^10,11^ structural degradation,^12,13^ gas evolution,^14,15^ and parasitic reactions with the electrolyte.^16,17^ However, the causality and connections between these different modes of degradation are not yet fully established. Oxygen loss from the cathode surface and the associated formation of a reduced surface layer have been widely observed for Ni-rich cathode materials particularly above the H2–H3 transition,^15,18–20^ and are found to depend critically on the upper cut off voltage (UCV) and electrolyte formulation.^17,21–23^ Recently, studies of Ni-rich cathodes at high potentials (≥4.3 V during charge) have also shown the emergence of a distinct signature in O K-edge resonant inelastic X-ray scattering (RIXS) spectra at an excitation energy of ∼531.5 eV.^24–26^ For Li-rich materials that show excess capacity beyond TM cation redox, this signature is typically taken as evidence of the formation of molecular O_2_ trapped in pores throughout the cathode bulk, as a result of charge compensation by non-bonding O orbitals.^27,28^ However, the TM vacancies necessary to accommodate this are not expected in LiNiO_2_, with experimental samples typically containing excess Ni. Furthermore, LiNiO_2_ does not exhibit excess capacity that might be associated with molecular O_2_ redox. Nevertheless, bulk sensitive Ni K-edge X-ray absorption Near Edge Structure (XANES) measurements of LiNiO_2_ have indicated a plateauing of the main edge half-height position at similarly high states of charge, which has been taken as evidence of the formal Ni oxidation state no longer changing in the bulk, and thus a change in the redox mechanism.^29^

Although O K-edge RIXS and the related fluorescence yield X-ray absorption spectroscopy (FY-XAS) are widely referred to as bulk-sensitive (∼200 nm information depth), in the context of Li-ion cathode materials where typical secondary particle diameters are >5 μm, these methods probe <10% of the particle volume nearest to the surface. Attribution of bulk molecular O_2_ redox based on these methods alone is therefore ambiguous. Similar concerns have been raised around identifying oxygen redox with hard X-ray photoelectron spectroscopy (HAXPES), where typical probing depths are tens of nm.^30^ Solid-state ^17^O magic-angle-spinning nuclear magnetic resonance (NMR) spectroscopy provides an alternative bulk-averaged approach to estimate the amount of O_2_ present in the lattice.^31^ However, it does not resolve the spatial distribution of O_2_ molecules, nor has it been reported for LiNiO_2_ to date. Whereas, online electrochemical mass spectrometry (OEMS) can quantify the gas release associated with O-loss from the cathode surface, it does not probe molecular O_2_ that remains trapped within the cathode.^15,20,32^ The extent to which oxygen redox is involved in charge compensation in the LiNiO_2_ bulk thus remains unclear, motivating approaches that can provide comparable information with surface- and bulk-sensitivity.

Here we combine complementary core-loss spectroscopies to obtain a depth-resolved (10 nm to >10 μm) account of the redox processes in LiNiO_2_ and distinguish reversible bulk redox processes from near-surface degradation. X-ray Raman Spectroscopy (XRS, >10 μm information depth) reveals that in the bulk of LiNiO_2_ secondary particles there is a continuous change in both the Ni L_3,2_-edge and O K-edge spectra with state of charge (SoC) up to 4.8 V. This is consistent with charge compensation proceeding by rehybridization between the Ni and O centres, lowering the electron density on O sites but with Ni–O coordination still preserved. Features of trapped molecular O_2_ appear at potentials of ≳4.2 V in O K-edge FY-XAS, accompanied by increased Ni^2+^ contributions in the Ni L_3,2_-edge. Importantly, these changes are less pronounced in bulk-averaged XRS measurements indicating that formation of molecular O_2_ is a predominantly surface process. Total Electron Yield (TEY)-XAS measurements (∼10 nm information depth) confirm that a densified NiO-like layer forms in direct contact with electrolyte, whilst FY-XAS measurements are consistent with an extended cation mixing layer in which Ni^2+^ ions have migrated to occupy Li sites. Scanning transmission electron microscopy–electron energy loss spectroscopy (STEM–EELS) further confirms this picture of a reduced surface layer (RSL) that extends ∼200 nm into the surface for LiNiO_2_ which has been cycled to 4.8 V *vs.* Li/Li^+^. This understanding emphasises the importance of strategies to stabilise the interfaces of Ni-rich cathode materials in contact with electrolyte (*e.g.* cathode coatings/gradients, electrolyte formulation), rather than bulk stabilisation approaches (*e.g.* pillaring) that might sacrifice capacity.

## Results and discussion

### Trends in bulk chemical state

**Fig. 1:**
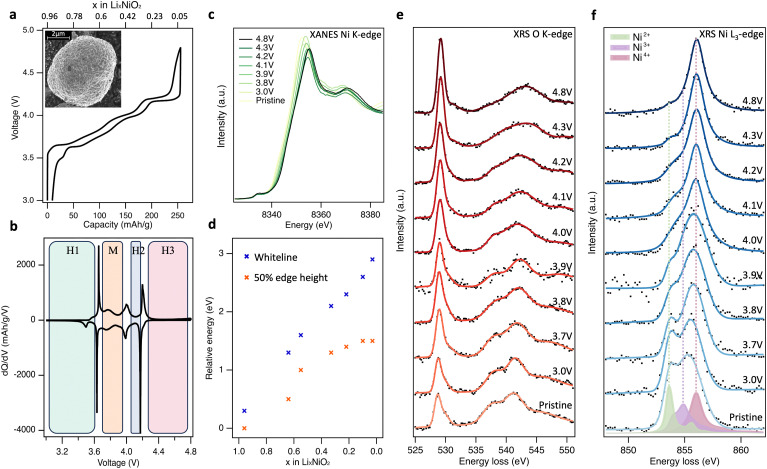
Bulk-sensitive probing of LiNiO_2_ redox processes. (a) 2nd cycle charge–discharge profile of LiNiO_2_ electrode cycled at a rate of C/20 between 3.0 and 4.8 V *vs.* Li/Li^+^. Inset: Scanning electron microscope (SEM) image of pristine LiNiO_2_ particles. (b) Corresponding differential capacity plots (d*Q*/d*V*). (c), Normalised Ni K-edge XANES spectra (transmission mode) of LiNiO_2_ at different SoC. (d) Plot of the energy shift in normalised Ni K-edge whiteline and 50% edge height positions relative to pristine LiNiO_2_. (e) and (f) XRS (∼10 μm information depth) of the O K-edge and Ni L_3_-edge core-loss spectra for LiNiO_2_ electrodes at different SoC during the 2nd charge cycle. Experimental XRS data is marked as black dots and represented with smooth solid trace lines. Charge transfer multiplet (CTM) calculations of formally Ni^2+^ (green), Ni^3+^ (purple), and Ni^4+^ (pink) environments. See ESI,† Fig. S2 for fitted XRS Ni L_3,2_-edges.

The charge–discharge profile for the 2nd cycle of the composite polycrystalline LiNiO_2_ electrode is shown in Fig. 1(a), together with an inset showing a scanning electron micrograph (SEM) of the pristine LiNiO_2_ active material (see ESI,† Fig. S1 for further characterisation). The ∼5 μm diameter spheroidal LiNiO_2_ secondary particles are composed of sub-μm primary particles. The voltage profiles show distinct plateaus associated with the first-order structural phase transitions of LiNiO_2_ on delithiation, apparent as maxima in the d*Q*/d*V* plots (Fig. 1(b)), at potentials consistent with prior literature.^15,33,34^ Powder X-ray diffraction (XRD) of the pristine material (see ESI,† Fig. S1c) closely resembles the calculated pattern for LiNiO_2_ with the *R*3̄*m* space group. This hexagonal H1 phase transitions to the monoclinic M phase at ∼3.67 V, then to the H2 phase at ∼4.0 V, followed by the H3 phase at ∼4.2 V. The voltage profile shows noticeable hysteresis above ∼4.3 V, with the voltage rapidly dropping from 4.8 V to ∼4.2 V on discharge. However, the capacity reached at 4.8 V is 256 mA h g^−1^ which compares with a maximum theoretical capacity of 264 mA h g^−1^, based on the pristine material having ∼4% Ni excess as determined by inductively coupled plasma-optical emission spectroscopy (ICP-OES).^26^ This provides an initial indication that the full capacity of the electrode can be accounted for by formal Ni redox alone, without obvious excess capacity associated with molecular O_2_ redox.

Fig. 1(c) shows normalised transmission Ni K-edge XANES spectra for the LiNiO_2_ electrodes at different SoC (*x* in Li_*x*_NiO_2_) during the 2nd charge cycle. As expected, the Ni K-edge shifts to higher energies as the formal Ni oxidation state increases, with the removal of valence electrons leaving the Ni nucleus less-shielded such that it has a higher effective charge, and the core-level becomes more strongly bound. Both the energy of the fractional (normalised) edge height and the position of the whiteline (intensity maximum) are routinely used as indirect measures of average oxidation state.^35,36^ A continuous shift to higher energy in both the edge half-height and whiteline is observed up to 4.2 V, *x* = 0.22 (Fig. 1(d)). The two trends diverge with further delithiation, with the whiteline monotonically shifting to higher energy up to the furthest measured extent of delithiation (4.8 V, *x* = 0.03), while the half-height position plateaus with little variation between *x* = 0.10 and *x* = 0.03. The plateau of half-height position has previously been taken as an indication that Ni is no longer involved in the redox mechanism at high SoC,^24,25^ however the continuing shift in whiteline position would suggest otherwise. Indeed, the edge-position is known to be sensitive to other factors including bond length and ligand covalency.^37^

To resolve this ambiguity without introducing surface sensitivity as a confounding factor, bulk-sensitive XRS was performed to collect O K-edge (Fig. 1(e)) and Ni L_3_-edge (Fig. 1(f)) spectra at the same SoC as the XANES. XRS probes lower-energy O 1s → 2p and Ni 2p → 3d transitions using hard X-rays (10 keV), achieving an information depth of ∼10 μm which is similar to Ni K-edge XANES. In Fig. 1(e), pristine LiNiO_2_ exhibits a prominent asymmetric O K pre-edge feature centred at 528.8 eV associated with transitions from O 1s → O 2p-Ni 3d hybridised states, and main edge features above 535.0 eV associated with transitions from O 1s → O 2p-Ni 4s,p hybridised states. On delithiation, the pre-edge peak is seen to continuously increase in relative intensity, whilst losing its asymmetry and shifting by 0.4 eV to a higher peak energy of 529.2 eV. There is also an accompanying shift in the main edge half-height position from ∼536.0 eV for pristine LiNiO_2_ up to 539.5 eV at 4.8 V, and the shape of the main edge changes indicating a change in the O2p and Ni4s,p orbital hybridisation. Importantly, across the potentials probed, the feature arising at ∼531.5 eV associated with the formation of molecular O_2_ is not strongly pronounced.^24–26^

The corresponding Ni L_3_-edge XRS (Fig. 1(f)) for pristine LiNiO_2_ shows a broad line shape composed of three main features at 853.6 eV, 854.9 eV, and 856.1 eV. There remains debate over the ground state of LiNiO_2_ (see Supplementary Note 1, ESI†) and a variety of models based on alternating layers of NiO_6_ octahedra and Li have been proposed. The simplest model, in which all NiO_6_ octahedra are equivalent with a formal oxidation state of Ni^3+^, is compatible with XRD data but not with measurements using more local probes.^38,39^ As a result, more complex models involving time or spatially varying distortions of the octahedra have been proposed. These include structures with Jahn–Teller (J–T) distortions, where two different Ni–O bond lengths are present and the formal oxidation state remains Ni^3+^,^40^ and spin disproportionated structures, where Ni^2+^ (*S* = 1), Ni^3+^ (*S* = ½), and Ni^4+^ (*S* = 0) octahedra coexist and interconvert dynamically at room temperature.^41,42^ Recent temperature-dependent XAS and X-ray magnetic circular dichroism (XMCD) shows strong evidence for such disproportionation in LiNiO_2_,^41^ which is consistent with other correlated nickelate compounds, including AgNiO_2_, which show disproportionation and strong covalency between frontier O 2p and Ni 3d states.^43–47^

Charge transfer multiplet (CTM) calculated L_3_-edges for the three Ni environments with formal oxidation states of +2, +3 and +4 are overlaid on the pristine LiNiO_2_ spectra in Fig. 1(f), corresponding to the three main features at 853.6 eV, 854.9 eV, and 856.1 eV seen in Ni L_3_-edge XRS. Simulation parameters have been optimised based on experimental data (Supplementary Note 3, ESI†). Each simulated spectra can be thought of as a superposition of metal–ligand hole configurations,^47^ with the formally Ni^2+^, Ni^3+^, and Ni^4+^ octahedra having ground-state configurations of 0.80|3d^8^〉 + 0.19|3d^9^L̲〉 + 0.01|3d^10^L̲^2^〉, 0.25|3d^7^〉 + 0.58|3d^8^L̲〉 + 0.16|3d^9^L̲^2^〉 + 0.01|3d^10^L̲^3^〉, 0.04|3d^6^〉 + 0.33|3d^7^L̲〉 + 0.48|3d^8^L̲^2^〉 + 0.14|3d^9^L̲^3^〉 + 0.01|3d^10^L̲^4^〉 respectively. In CTM calculations, increasing ligand hole contributions indicate an increasing degree of Ni–O covalency for higher formal oxidation states. Linear combinations of the simulated spectra match closely to the Ni L_3_-edge spectra from XRS, FY-XAS and TEY-XAS at all SoC (ESI,† Fig. S2–S5), indicating that the simulated spectra for the Ni^2+^, Ni^3+^ and Ni^4+^ environments are suitable descriptions despite the small changes in octahedral environment expected for different phases.

**Fig. 2:**
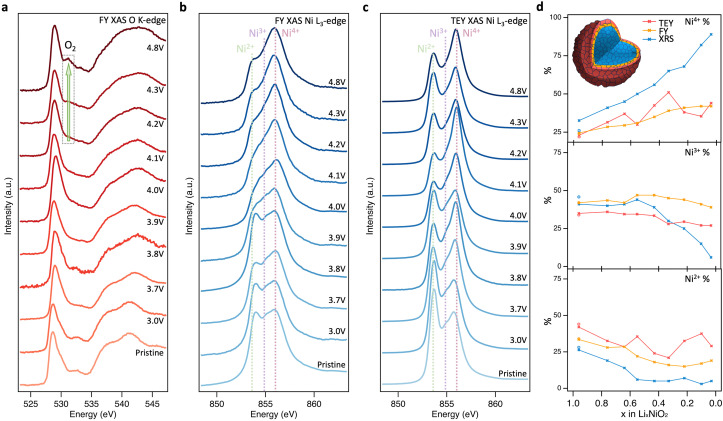
Near-surface probing of LiNiO_2_ redox processes. (a) and (b) FY-XAS (∼200 nm information depth) of the O K-edge and Ni L_3_-edge, and (c) TEY-XAS (∼10 nm information depth) of the Ni L_3_-edge for LiNiO_2_ at different SoC. (d) Relative intensities of Ni^2+^, Ni^3+^, and Ni^4+^ components based on fitting CTM calculated spectra to XRS, FY-XAS, and TEY-XAS spectra (see fitting results in ESI,† Fig. S2–S5).

On cycling to higher potentials, the XRS shows a continuous growth in the intensity of the Ni^4+^ feature (see Fig. 2(d)), initially at the expense of Ni^2+^ up to 3.9 V, *x* = 0.55, and then Ni^3+^ up to 4.8 V, *x* = 0.03. This evolution of Ni species upon delithiation matches that expected from disproportionation.^41^ At 4.8 V, the spectrum closely matches Ni L_3_-edge simulations of Ni^4+^ (ESI,† Fig. S6) with 4–5% Ni^2+^. This is consistent with the excess Ni detected with ICP-OES occupying Li sites, as similarly sized Ni^2+^, and thus preventing all sites reaching Ni^4+^.^26,48^ The bulk sensitivity of XRS suppresses contributions from surface layers which are otherwise seen even for inverse partial fluorescence yield (IPFY) measurements (ESI,† Fig. S7), including for reference Ni^4+^ compounds.^49,50^ Importantly this shows that charge compensation in the LiNiO_2_ bulk proceeds predominantly through Ni–O rehybridization across the whole cycling range, lowering the electron density on O sites, but without a significant role for molecular O_2_ redox. This contrasts with several reports of oxygen redox in this potential range for LiNiO_2_ and Ni-rich layered cathode materials, based on detection of the molecular O_2_ feature with less bulk-sensitive O K-edge RIXS.^24–26,51^

### Near-surface degradation

To further investigate the origins of molecular O_2_ reported at high SoC, the same core levels were measured using soft XAS in FY mode (Fig. 2(a) and (b)). The spectra for pristine LiNiO_2_ closely resemble those obtained with XRS, however an additional feature is apparent at 532.3 eV in the O K-edge, and the Ni^2+^ feature in the Ni L_3_-edge is more intense. These same features are seen for NiO (see ESI,† Fig. S8 for O K-edge), and correspond to a NiO-like RSL,^52^ whose contribution is not detected in the more bulk-sensitive XRS. On delithiation, the XAS data show similar trends to the XRS until 4.1 V, *x* = 0.33, with the Ni L_3_-edge showing the Ni^4+^ feature increasing at the expense of Ni^2+^ and then Ni^3+^, and some growth in the O K pre-edge. At higher SoC there are significant deviations between FY-XAS and XRS spectra. Most notably a feature at ∼531.5 eV is seen to emerge in the O K-edge, which although initially weak at 4.2 V, *x* = 0.22, shows significant intensity at 4.8 V, *x* = 0.03 (see integrated peak areas in ESI,† Fig. S9). This feature corresponds to the same absorption energy as molecular O_2_, whose vibrational structure has been detected in LiNiO_2_ and other conventional Ni-rich layered oxides in several recent reports.^25,26^ Whereas this molecular O_2_ signature and the Ni^2+^ feature grow in FY-XAS, the O K pre-edge peak and the Ni^4+^ feature in the Ni L_3_-edge are supressed in FY-XAS compared to the XRS. This suggests a near-surface molecular O_2_ redox process associated with RSL growth, in which Ni is reduced toward Ni^2+^ and molecular O_2_ forms *i.e.*, NiO_2_ → NiO_2−*x*_ + ½*x*O_2_. Similar trends are observed with the more surface-sensitive TEY-XAS (fits shown in ESI,† Fig. S4) consistent with RSL formation proceeding from electrolyte-exposed surfaces.^21,52^ Importantly, the bulk-sensitive XRS (Fig. 1(e) and (f)) does not detect such Ni reduction or O_2_ formation, even after charging to 4.8 V, highlighting the key connection between the formation of trapped molecular O_2_ and the increase in Ni^2+^ species close to the cathode surface.

**Fig. 3:**
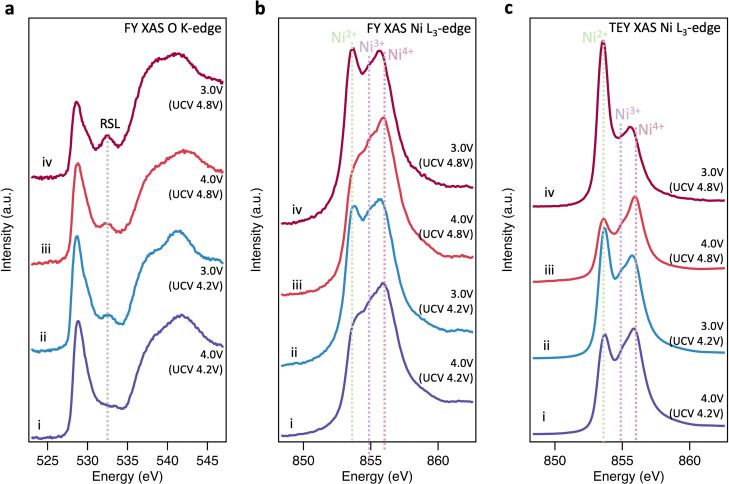
Discharge behaviour of LiNiO_2_. (a), (b) FY-XAS (∼200 nm information depth) of the O K-edge and Ni L_3_-edge, and (c) TEY-XAS (∼10 nm information depth) of the Ni L_3_-edge for LiNiO_2_ cycled to a UCV of 4.2 V before being discharged to (i) 4.0 V and (ii) 3.0 V, with parallel samples cycled to a higher UCV of 4.8 V and then back to (iii) 4.0 V and (iv) 3.0 V.

We now investigate the reversibility of this near-surface molecular O_2_ redox process and how its extent changes with upper cutoff voltage (UCV). Fig. 3(a) shows that after discharging from a UCV of 4.8 V to 4.0 V, the molecular O_2_ feature at ∼531.5 eV disappears from the O K-edge, but a prominent RSL feature at 532.6 eV remains. On discharge to 3.0 V, the RSL feature further grows in intensity relative to the pre-edge feature, with accompanying increases in the Ni^2+^ feature for the Ni L_3_-edge spectra (Fig. 3(b) and (c)). This is even more prominent in the surface-sensitive TEY-XAS (Fig. 3(c)), indicating the RSL is more densified near to the surface. Comparison to an electrode where the UCV is 4.2 V confirms that the extent of RSL formation is much greater for the UCV of 4.8 V, consistent with previous studies where significant RSL formation occurs at SoC above the H2-H3 transition in Ni-rich cathodes.^20,21,52^ Longer-term cycling (150 cycles, ESI,† Fig. S10) further shows that the UCV of 4.8V leads to greater voltage hysteresis and charge transfer impedance reflecting this more extensive RSL formation.

**Fig. 4:**
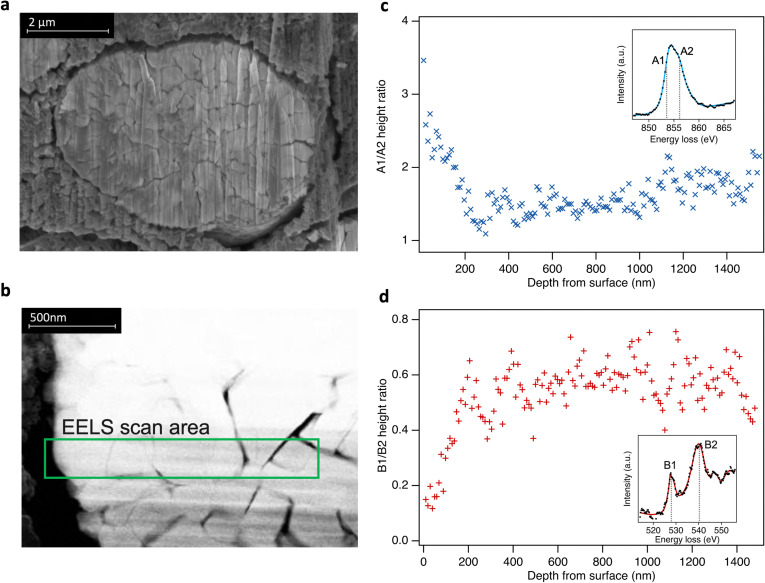
(a) Cross-sectional scanning electron microscopy (secondary electron detection) of LiNiO_2_ particle from an electrode charged to 4.8 V. (b) Selected STEM-EELS scan area of 1.5 μm from surface to bulk (left to right) of the particle. (c) and (d) Fitted peak ratios of depth-resolved Ni L_3_- and O K-edge EELS spectra using a simplified two peak fit in each case (see ESI,† Fig. S11). Insets: Examples of EELS spectra.

Although comparison of TEY and FY mode XAS confirms the RSL is found predominantly near the sample surface, it provides only limited insight into the depth over which it is distributed. To spatially resolve the extent of the RSL at high SoCs, STEM-EELS was performed for LiNiO_2_ charged to 4.8 V (Fig. 4). Depth-resolved Ni L_3_-spectra show a decreasing proportion of the lower energy (more reduced) component (peak A1) on moving towards the bulk of the particle, stabilising at ∼200 nm from surface, consistent with more Ni^2+^ species at the surface and more Ni^4+^ in the bulk. Similarly, the O K-edge shows a higher pre-edge intensity (peak B1) towards the bulk of the particle correlating with higher Ni oxidation state and Ni–O covalency. This extended RSL region where the Ni oxidation state is seen to vary over ∼200 nm is attributable to a cation mixing layer in which Ni^2+^ ions have migrated to occupy Li sites, and is consistent with the differences seen between TEY-XAS, FY-XAS and XRS observations. Notably, a similar extent of RSL formation is not observed at intergranular cracks away from the LiNiO_2_ surface, presumably as electrolyte does not fully penetrate these cracks for the low cycle numbers considered here. This indicates a key role of the electrolyte in promoting RSL formation, with electrolyte infiltration into internal cracks likely proceeding over multiple cycles.

### Bulk electronic and geometric structural evolution of LiNiO_2_

**Fig. 5:**
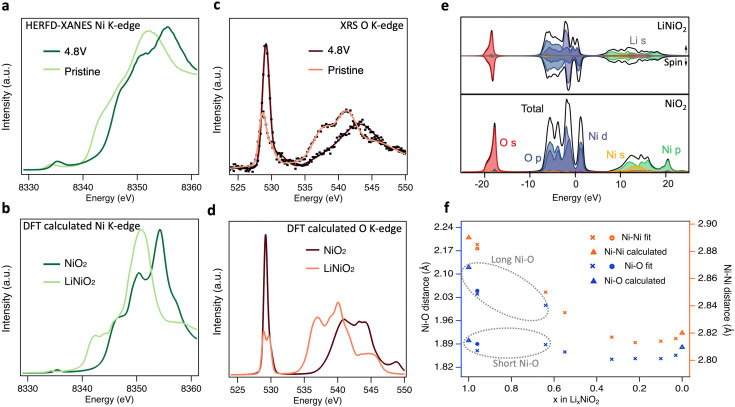
Electronic and geometric structural changes of LiNiO_2_ upon delithiation. (a) Experimental HERFD-XANES and b, core–hole calculated Ni K-edge spectra of pristine and charged LiNiO_2_. (c) Experimental XRS with smooth trace lines and (d) core–hole calculated O K-edge spectra of pristine and charged LiNiO_2_. (e) Ground-state partial and total density of states for LiNiO_2_ (top) and NiO_2_ (bottom). Fermi energies are set to zero. (f) Ni–Ni and Ni–O distances determined from the Fourier-transformed EXAFS spectra (details in ESI,† Table S1 and Fig. S12, S13). Note that the short/long Ni–O lengths of pristine (hexagons), 3.0 V and 3.8 V (crosses) LiNiO_2_ are related to the disproportionated model applied for EXAFS fitting. Bond lengths for the geometry optimised structures from DFT calculations used in (b), (d), (e) are shown as triangles in f.

Having shown that bulk redox in LiNiO_2_ occurs by Ni–O rehybridization, we now consider further the associated changes in electronic and geometric structure. Fig. 5(a) shows high-energy-resolution fluorescence detection (HERFD-)XANES Ni K-edge spectra of pristine LiNiO_2_ and after cycling to 4.8 V. Notably the main edge half-height position is shifted ∼2.1 eV higher compared to LiNiO_2_, a more distinct change than seen in the transmission mode measurements of Fig. 1(c) (∼1.5 eV), as a result of the fine-structure features along the rising edge now being better resolved. Fig. 5(c) compares the similarly bulk-sensitive O K-edge XRS spectra of the same samples. Since we anticipate that differing Ni–O bond lengths yield distinct signatures in the O K pre-edge in any model of the material, we chose the zigzag J–T *P*2_1_/*c* structure for LiNiO_2_ spectral calculations. More complex time-varying LiNiO_2_ model structures are computationally prohibitive for spectral calculations and although the *P*2_1_/*c* structure has a formal oxidation state of Ni^3+^, this will predominantly affect the Ni 3d states, which only weakly influence the main features of the Ni and O K-edge spectra. Density functional theory (DFT) calculated Ni and O K-edge spectra for LiNiO_2_ and NiO_2_ (Fig. 5(b) and (d)), reproduce the features of the experimental spectra extremely well, showing the same pre-edge peaks, number of fine structure features, and similar trends in intensity and linewidths across the whole spectral range. The relative energy shifts are also captured well, giving confidence in the sufficiency of the chosen structure models (*P*2_1_/*c* for LiNiO_2_, and *R*3̄*m* for NiO_2_).

The origin of the spectral features can be understood by comparison to ground-state partial density-of-states (pDOS) shown in Fig. 5(e), and consideration of the allowed spectroscopic transitions. The first unoccupied states in both LiNiO_2_ and NiO_2_ lie just above 0 eV, showing mixed O 2p and Ni 3d orbital character and giving rise to the pre-edge peaks in the experimental Ni (∼8335 eV) and O (∼529 eV) K-edges. A sizable gap separates the next set of unoccupied states which give rise to the main edges in the Ni (≳8340 eV) and O (≳535 eV) K-edges, and have Ni 4s,p character, with some Li 2s contribution also seen in this region for LiNiO_2_. This gap widens by ∼2.9 eV from LiNiO_2_ to NiO_2_ which can be related to a decrease in average Ni–O bond length associated with the change in geometric structure.^53,54^ We note that the DFT calculated Ni K-edge spectra show weaker pre-edge features than experiment, attributable to quadrupolar transitions not being considered in the calculations.^55^

A clear splitting of the calculated O K pre-edge peak in Fig. 5(d) for LiNiO_2_ resembles the asymmetric pre-edge in the XRS experimental data. The O K pre-edge becomes far more intense in the 4.8 V sample and the peak splitting seen in the calculated spectrum of LiNiO_2_ is lost. This corresponds with the change from *D*_4h_ site symmetry for the J–T distorted Ni^3+^ octahedra used in the LiNiO_2_ calculation, where d orbital splitting arises from the elongation of two Ni–O bonds, to O_h_ site symmetry for the Ni^4+^ octahedra of NiO_2_, where this d orbital splitting is lost. The growth in intensity of the O K pre-edge feature is also consistent with the CTM calculations, where the increased ligand hole contributions for the Ni^4+^ octahedra indicate an increasing degree of Ni–O covalency on delithiation. The increase of O K pre-edge intensity by a factor of ∼2 on full delithiation (see ESI,† Fig. S9) corresponds closely to the factor of ∼1.8 obtained based on the proportions of Ni species fitted to the Ni L_3,2_-edge XRS spectra (Fig. 2(d)) and their respective electron configurations. Further evidence for increased Ni–O covalency is apparent from the emergence of more distinct fine-structure features (∼8347 eV and 8351 eV) in the Ni K-edge, attributable to ligand-to-metal charge transfer shakedown transitions,^56^ as well as satellite peaks in the Ni L_3,2_-edge that are most clearly seen in FY-XAS measurements (see ESI,† Fig. S14b) and are well-reproduced in the CTM calculated Ni^4+^ spectrum. In addition, Bader charge analysis^57^ based on the ground-state DFT calculations shows the ionic charge of the Ni only modestly changes from +1.41 to +1.56 *e*^−^ between LiNiO_2_ and NiO_2_, whilst a more significant change from −1.15 to −0.78 *e*^−^ is seen for the O charges.

Fig. 5(f) shows the nearest Ni–O and Ni–Ni distances extracted by fitting to EXAFS spectra for LiNiO_2_ at different SoC. Since fitting with the single Ni–O bond length model showed significantly higher Debye–Waller factors at low SoC, and disproportionation is expected to persist up to 3.9 V based on Fig. 2(d), a model with two Ni–O bond lengths (ratio of short:long Ni–O bond based on the disproportionated model and associated XRS fittings) was instead used to fit pristine, 3.0 V and 3.8 V LiNiO_2_ (see ESI,† Fig. S15 and Table S2). The Ni–O and Ni–Ni bond distances obtained show good agreement with both the J–T *P*2_1_/*c* LiNiO_2_ and the disproportionated structure, however, the short:long bond ratios of the disproportioned model show lower Debye–Waller factors, supporting assignment of this structure.

Similar trends in weighted average Ni–O bond lengths are seen to *operando* neutron diffraction measurements,^34^ with Ni–O bond length gradually shrinking in line with the change in structure, increased oxidation state and increased covalency at high SoC. Notably, above the H2–H3 transition (*x* ≤ 0.22) a modest increase in the Ni–O bond length is observed. This has been associated with a loss of the stabilising effect of Li–O covalency at high SoC, leading to Ni–O bond elongation alongside the sudden *c*-lattice collapse related to the H2–H3 transition, and increased charge transfer from the O to Ni sites.^11,58,59^ This changing covalency, seen as continuous spectral changes in Fig. 1(c) and (d), can account for the plateauing in half-height position of the Ni K main-edge at high SoC in transmission XANES (Fig. 1(d)). As well as highlighting the limitations of applying a single metric to assess changes in oxidation state, the limited sensitivity of the Ni K-edge fractional-edge height reflects that it arises from transitions to Ni 4s,p states, in contrast to the O K- and Ni L_3,2_-edges which probe transitions to O 2p-Ni 3d hybridised states.

## Conclusion

**Fig. 6:**
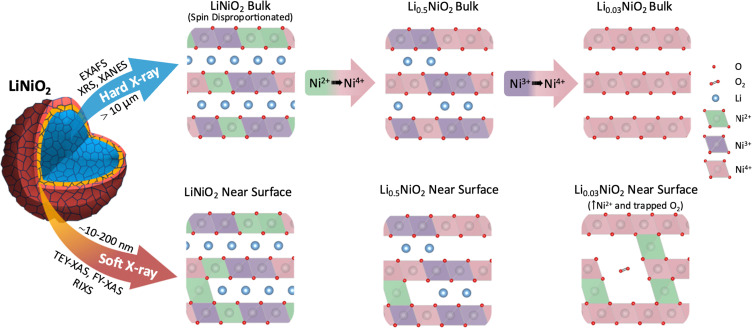
Schematic representation of LiNiO_2_ bulk charge compensation mechanism and surface degradation processes probed by different X-ray spectroscopy techniques. The differences in LiNiO_2_ delithiation occurring at the surface and in the bulk, including the accommodation of trapped O_2_ in pores formed near the surface by Ni^2+^ species migrating to the Li layer. Continuous oxidation of Ni in bulk LiNiO_2_ at high SoC is distinguished from the near surface degradation.

In summary, bulk sensitive XRS measurements reveal that in the bulk of LiNiO_2_, charge compensation occurs by Ni–O rehybridization without the involvement of molecular O_2_. From an initially disproportionated structure where formally Ni^2+^, Ni^3+^, and Ni^4+^ octahedra coexist, the Ni^4+^ features of the Ni L_3,2_-edge continuously grow on delithiation, initially at the expense of Ni^2+^ and subsequently Ni^3+^ features (see Fig. 6). There is a concomitant increase in O K pre-edge intensity, consistent with significant lowering of electron density on the O sites at potentials where O loss is expected.^9,50^ However, significant signatures of molecular O_2_ formation are not detected throughout the bulk suggesting its formation remains kinetically hindered.^60^

FY-XAS measurements reveal evidence of molecular O_2_ formation in the outer ∼200 nm of the cathode surface, with the growth in intensity of the Ni^4+^ feature plateauing above 4.2 V, and features of trapped molecular O_2_ emerging alongside increased Ni^2+^ contributions. This is consistent with molecular O_2_ trapped in voids formed by Ni^2+^ entering the Li layers (see Fig. 6). STEM-EELS reveals a RSL that extends ∼200 nm into the LiNiO_2_ surface following cycling to 4.8 V, showing a gradual change in oxidation state across its thickness. The absence of this extended RSL at internal surfaces of the secondary cathode particles, *e.g.* interparticle cracks, suggests its formation is driven by contact with the electrolyte.

The trapped molecular O_2_ feature disappears on discharging to 4.0 V, but a significantly increased near-surface Ni^2+^ contribution is retained. Although our results do not fully exclude some reversible molecular O_2_ redox, online electrochemical mass spectroscopy studies have reported O_2_ evolution occurring on discharge.^15^ We therefore suggest that this may arise from the release of trapped O_2_ associated with structural changes, including abrupt *c*-lattice expansion and particle cracking (ESI,† Fig. S16).

Our findings highlight the importance of combining bulk- and surface-sensitive techniques to fully confirm the extent to which molecular O_2_ redox processes in cathode materials are bulk phenomena contributing to reversible charge compensation, rather than involved in surface degradation as revealed here for LiNiO_2_. The understanding developed of the surface instability of LiNiO_2_ associated with rehybridisation at high SoC, emphasises the importance of strategies such as cathode coatings, composition gradients, and electrolyte formulation to stabilise Ni-rich cathode surfaces in contact with electrolyte, rather than bulk stabilisation approaches (*e.g.* pillaring) that might unduly sacrifice capacity. This study thus provides a solid basis for future exploration of molecular O_2_ formation and Ni–O rehybridisation in Ni-rich cathodes in different electrolyte environments, and for further investigations to separate bulk redox and near-surface degradation processes in a broad range of cathode materials.

## Experimental

### Sample preparation

Commercial grade LiNiO_2_ powder was obtained from BASF, without any deliberate doping or coating added. This was characterised by SEM (Zeiss Merlin, 2 kV, Inlens detector), XRD (Rigaku Miniflex, Cu Kα source), and X-ray Photoelectron Spectroscopy (XPS, PHI Versaprobe III, Al Kα source), see ESI,† Fig. S1. Free-standing electrodes were prepared by calendaring a mixture of 80 wt% LiNiO_2_ powder, 10 wt% conductive acetylene black and 10 wt% polytetrafluoroethylene (PTFE) binder. Electrochemical tests of the LiNiO_2_ cathodes were performed in 2032 coin cells (316 stainless steel, Cambridge Energy Solutions) using Li metal disks as negative electrodes and borosilicate glass fibre separators (borosilicate, GF/A, Whatman) soaked with 120 μL of LP57 electrolyte (1 M LiPF6 in 3 : 7 of EC : EMC). The assembled cells were charged up to 4.2 V at C/20 (calculated based on a theoretical capacity of LiNiO_2_ of 275 mA h g^−1^), held for 30 min and then cycled back to 3.0 V at the same rate. This was immediately followed by a second charge at C/20 (ESI,† Fig. S1), with a voltage hold for 10 hours at the desired endpoint. The SoC of delithiated Li_*x*_NiO_2_ is calculated based on the charge–discharge capacity curve presented in Fig. 1(a), starting from *x* = 0.96 for pristine and 3.0 V LiNiO_2_ (based on the ∼4% Ni excess). All potentials mentioned in this work are referenced to Li/Li^+^. For *ex situ* measurements, cathodes were recovered from the cycled coin cells by disassembly in a glove box under Ar atmosphere (O_2_ < 1 ppm, H_2_O < 1 ppm). Recovered cathodes were washed in dimethyl carbonate (anhydrous, ≥99%, Sigma Aldrich) solvent and dried before heat-sealing in aluminised mylar (Fresherpack, 130 μm) pouches for XANES, EXAFS, and XRS, or transferring in a vacuum suitcase for XAS, XPS.

### Electron microscopy

A Thermo Scientific Helios G4 CXe Plasma FIB (PFIB) was used to prepare the STEM lamellae. For lift-out, a thin Pt layer was deposited onto the surface region of interest (ROI), trenches were patterned around the ROI to make 4 μm-thick lamellae. A W needle was then used to lift each lamella and place them on a FIB lift-out grid (Cu, Agar Scientific). Each lamellae was then thinned down to around 50 nm thick for STEM-EELS and polished with a low dose, low energy beam (<0.3 nA, 5 keV) to minimise ion beam damage. Inert transfer between PFIB and an Ar glovebox was achieved using a Gatan iLoad system.

Spatially resolved EELS of the lamellae was performed using a JEOL ARM200F equipped with cold field emission gun operated at 200 keV and spherical aberration probe corrector. Dual EELS was acquired using a Gatan GIF Quantum 965 ER with energy resolution of around 1 eV at 0.25 eV per channel dispersion. Inert transfer between glovebox and STEM was achieved using a JEOL double-tilt vacuum transfer holder.

Given LiNiO_2_ is less stable when highly delithiated, radiation damage should be considered when evaluating the oxidation state of Ni in EELS. The energised Xe ion beam in PFIB and electron beam in STEM can both induce reduction of LiNiO_2_ and NiO_2_ towards NiO.^61^ The absolute A2/A1, and B2/B1 ratios seen in the LiNiO_2_ bulk reflect some degree of ion/electron beam induced reduction. Nevertheless, equal acquisition time and constant electron beam current during the EELS scans ensure a consistent radiation dose (3 × 10^3^ e^−^ Å^−2^) such that the trends in EELS spectra and the spatial variations seen near their surface are still representative.

### X-ray spectroscopy

Transmission Ni K-edge XANES and EXAFS spectra were collected with a laboratory-based easyXAFS300+ spectrometer (easyXAFS, WA, US). X-rays are generated with a liquid-cooled Ag anode X-ray tube, before monochromation by a Si (551) spherically bent crystal analyser. A helium-filled box with polyimide windows is placed in the beam path for better X-ray transparency while a steel plate with a 9 × 3 mm slot is placed after each sample to lower the background. The transmitted intensity is measured with a silicon drift detector (KETEK, Munich, Germany) placed behind the sample. Each acquisition was performed over 45 min and 30 scans were collected for each sample to obtain good statistics. NiO reference spectra were also collected for each batch of measurements for energy calibration. Data pre-processing was performed with the EasyXANES package to convert the measured intensity into linear attenuation coefficient, *μ*. Data reduction and analysis were performed using the Demeter package (version: 0.9.26).

*In situ* HERFD-XANES was performed at Diamond Light Source's beamline I20 with aluminised mylar (Fresherpack, 130 μm) pouch cells containing free-standing LiNiO_2_ cathodes, with Li metal disks as negative electrodes and a Celgard 2325 separator soaked with 80 μl of LP57 electrolyte (1 M LiPF6 in 3 : 7 of EC : EMC). The cells were held at the desired potentials, and measured through the cathode side of the pouches using an X-ray emission spectrometer equipped with three Si(444) analyser crystals.^62^ The spectrometer was set to the maximum of the Ni Kβ_1,3_ line (8266 eV), and the incident energy was scanned using the four-bounce Si(111) monochromator. The spectrometer was calibrated using a Ni foil, measuring the Kβ line with the incident energy tuned +300 eV from the Ni K-edge.

TEY- and FY-XAS measurements were performed at ES-2 of beamline B07-B at Diamond Light Source, with the exit slits set to 50 μm in the dispersive direction, yielding a flux of between 1 × 10^11^ (O K-edge) and 2 × 10^11^ (Ni L_3,2_-edge) photons s^−1^. All samples were measured with the incident beam normal to the electrode surface, yielding a beam footprint of 150 × 100 μm. FY-XAS measurements were acquired using an Al coated Si photodiode directed at the sample with its surface normal at ∼45° to incident beam direction. Simultaneous TEY-XAS measurements were obtained using a SR570 low-noise current amplifier (Stanford Research Systems) to collect the current between the sample plate and an isolated steel washer in front of the sample biased to +90 V. Separate IPFY-XAS measurements of the Ni L_3,2_-edge were acquired using a Vortex silicon drift detector (Hitachi) at the I10 beamline at Diamond Light Source, with FY and TEY mode measurements simultaneously acquired. All spectra are divided by the drain current measured from the last X-ray mirror, to correct for variations in incident photon flux. The photon energy scale is calibrated using a NiO sample.^63^ O K-edge spectra are background-subtracted using a straight line fitted to the pre-edge region, followed by intensity normalization to the post-edge region at 550 eV. Ni L_3,2_-edge spectra are normalized to the intensity at 867 eV after removal of a linear background.

XRS measurements were performed at the European Synchrotron Radiation Facility at the ID20 beamline.^64^ X-rays are generated from three U26 revolver undulators, before being collimated, and then monochromated by a liquid–nitrogen cooled double-crystal Si(111) pre-monochromator. The beam from a second Si(311) channel-cut post-monochromator is focussed onto a ∼20 × 20 μm^2^ spot at the sample position by a mirror in Kirkpatrick–Baez geometry. The sample surface was positioned at a grazing angle of ∼1° relative to the incident beam direction, to maximise the illuminated area and the sample was scanned over a region of ∼10 mm during the 4-hour measurement to minimise beam-induced changes. Inelastically scattered photons were recorded using 72 spherically bent Si(660) crystal analysers with energy loss events in the vicinity of both O K-edge and Ni L_3,2_-edge. O K- and Ni L_3,2_-edges were recorded at momentum transfers of *q* = 6.9 ± 0.5, and all data extraction and treatment were performed as described in ref. 65.

### Charge-transfer multiplet calculations

Ni L_3,2_-edge multiplet simulations were performed at the ligand field level of theory using the many-body code, Quanty.^66^ This was implemented using the same single-cluster NiO_6_ Hamiltonian as Green *et al.*,^47^ where Ni 2p, Ni 3d ligand shells are explicitly included (see Supplementary Note S3, ESI†). Parameters used in Ni^2+^ calculation (eV): *Δ* = 5.5, 10*D*_q_ = 0.71, *V*_eg_ = 2.627, *V*_t2g_ = 1.524. Parameters used in Ni^3+^ calculation (eV): *Δ* = −0.5, 10*D*_q_ = 0.93 with Jahn–Teller splitting of *Δ*_eg_ = 0.15 and Δ_t2g_ = 0.10 where *Δ*_eg_ is the difference between the *x*^2^ − *y*^2^ and 3*x*^2^ − *r*^2^ onsite energies and Δ_t2g_ is the difference between the *xy* and *xz*/*yz* onsite energies, *V*_3*x*^2^ − *r*^2^_ = 2.43, *V*_*x*^2^ − *y*^2^_ = 3.33, *V*_*xz*/*yz*_ = 1.41, *V*_*xy*_ = 1.93. Parameters used in Ni^4+^ calculation (eV): *Δ* = −6.5, 10D_q_ = 0.78, *V*_eg_ = 3.456, *V*_t2g_ = 2.004.

### DFT spectral calculations

Density functional theory (DFT) calculations were carried out using the plane wave pseudopotential code CASTEP^67^ and the Perdew–Burke–Ernzerhof (PBE) form of the generalized gradient approximation functional,^68^ with the addition of the G06 semi-empirical dispersion correction^69^ to better account for van der Waals forces. The zig-zag J–T *P*2_1_/*c* structure for LiNiO_2_, and the *R*3̄*m* structure for NiO_2_ were used for pristine LiNiO_2_ and fully delithiated materials respectively.^33^ Each structure was initially geometry optimised using appropriate plane wave cut-off energies (900 eV) and *k*-points (0.03 Å^−1^*k*-point spacing) determined *via* convergence of the total energy. The geometry of the system was considered optimized when the maximum forces on the ions were below 0.001 eV Å^−1^ for NiO_2_ and 0.01 eV Å^−1^ for LiNiO_2_ consistent with other studies.^33,70^ Calculations of the pDOS and core–hole spectra were subsequently performed. The energy scale of the ground-state pDOS assumes the material is an insulator and sets the Fermi energy, *E*_f_, to zero. Since core orbitals are not treated explicitly in the pseudopotential method, a unique pseudopotential is generated for an excited atom possessing a core–hole. For O and Ni K-edges, a core–hole is placed on the O 1s or Ni 1s orbitals respectively. A supercell is generated to prevent interactions between neighbouring core–holes. For spectral calculations, the plane wave energy cut-off, *k*-point sampling and cell size were increased until no visible effect on the spectrum was seen. Spectral calculations were handled using the OptaDOS programme.^71^ Lorentzian broadening was performed using full widths at half maximum of 0.14 and 0.8 eV for the O and Ni K-edges respectively, which should reflect the lifetimes of radiative and non-radiative transitions.^72,73^ The Gaussian component was then adjusted as a free parameter to match the experimental data, but remained fixed for the same edges to allow for comparison. The Lorentzian component is given energy dependence to account for the energy dependence of the lifetime. This was done by summing the set width with a factor that varies linearly with energy as implemented in Optados. The calculated spectra were rigidly shifted to align with the first absorption peaks of the experimental data to allow better comparison. In cases where the system under investigation possessed more than one inequivalent excitation site, separate spectra were generated, energy aligned^74^ and combined before rigidly shifting.

## Author contributions

LA, JENS and RSW conceived the study. LA performed electrode preparation and electrochemical testing, with assistance from RAH. MWF performed SEM. LA, JENS, CJS and RSW performed the XRS. LA, AP, EB, PND, MWF, LAHJ, CMEP and RSW performed the soft XAS with support from DCG, PF and PB. LA, JENS, AP, CJS, and RSW performed XAS and XRS analysis. LA and PC performed the lab-based XANES and EXAFS, with analysis performed by PC. LA, JENS, EB, PND and RSW performed HERFD-XANES with support from SH. JENS, NR and RJN performed DFT calculations. RZ and PDN performed PFIB, STEM-EELS and related analysis. GH and RJG performed CTM calculations. LA, JENS, PC and RSW wrote the paper with contributions from all authors.

## Data availibility

The data supporting this article have been included as part of the ESI.† The corresponding data sets are available from the ORA repository, https://doi.org/10.5287/ora-yxpnqgero.

## Conflicts of interest

The authors declare that there are no conflicts of interest.

## Supplementary Material

EE-017-D4EE02398F-s001
