# Supplementary material for: Distinguishing bulk redox from near-surface degradation in lithium nickel oxide cathodes
Source: Energy Environ Sci. 2024 Sep 13;17(21):8379–91. doi: 10.1039/d4ee02398f (PMC11462161; doi:10.1039/d4ee02398f)
Supplement: EE-017-D4EE02398F-s001 [file EE-017-D4EE02398F-s001.pdf]

Supplementary Information (SI) for Energy & Environmental Science.
This journal is © The Royal Society of Chemistry 2024

Supplementary Information: Distinguishing bulk redox from near-surface degradation in lithium nickel oxide cathodes

Lijin An[1], Jack E. N. Swallow[1], Peixi Cong[1], Ruomu Zhang[1], Andrey D. Poletayev[1,2], Erik Björklund[1,2], Pravin N. Didwal[1,2], Michael W. Fraser[1,2], Leanne A. H. Jones[1], Conor M. E. Phelan[1], Namrata Ramesh[1], Grant Harris[3], Christoph J. Sahle[4], Pilar Ferrer[5], David C. Grinter[5], Peter Bencok[5], Shusaku Hayama[5], M. Saiful Islam[1,2], Robert House[1,2], Peter D. Nellist[1,2], Robert J. Green[3,6], Rebecca J. Nicholls[1], Robert S. Weatherup[1,2,5,]*

1 Department of Materials, University of Oxford, Parks Road, Oxford OX1 3PH, UK

2 The Faraday Institution, Quad One, Harwell Science and Innovation Campus, Didcot OX11 0RA, U.K.

3 Department of Physics and Engineering Physics, University of Saskatchewan, Saskatoon, Canada S7N 5E2

4 ESRF – The European Synchrotron, 71 Avenue des Martyrs, 38000 Grenoble, France

5 Diamond Light Source, Harwell Science and Innovation Campus, Didcot OX11 0DE, U.K.

6 Stewart Blusson Quantum Matter Institute, University of British Columbia, Vancouver, Canada V6T 1Z1

[*] robert.weatherup@materials.ox.ac.uk

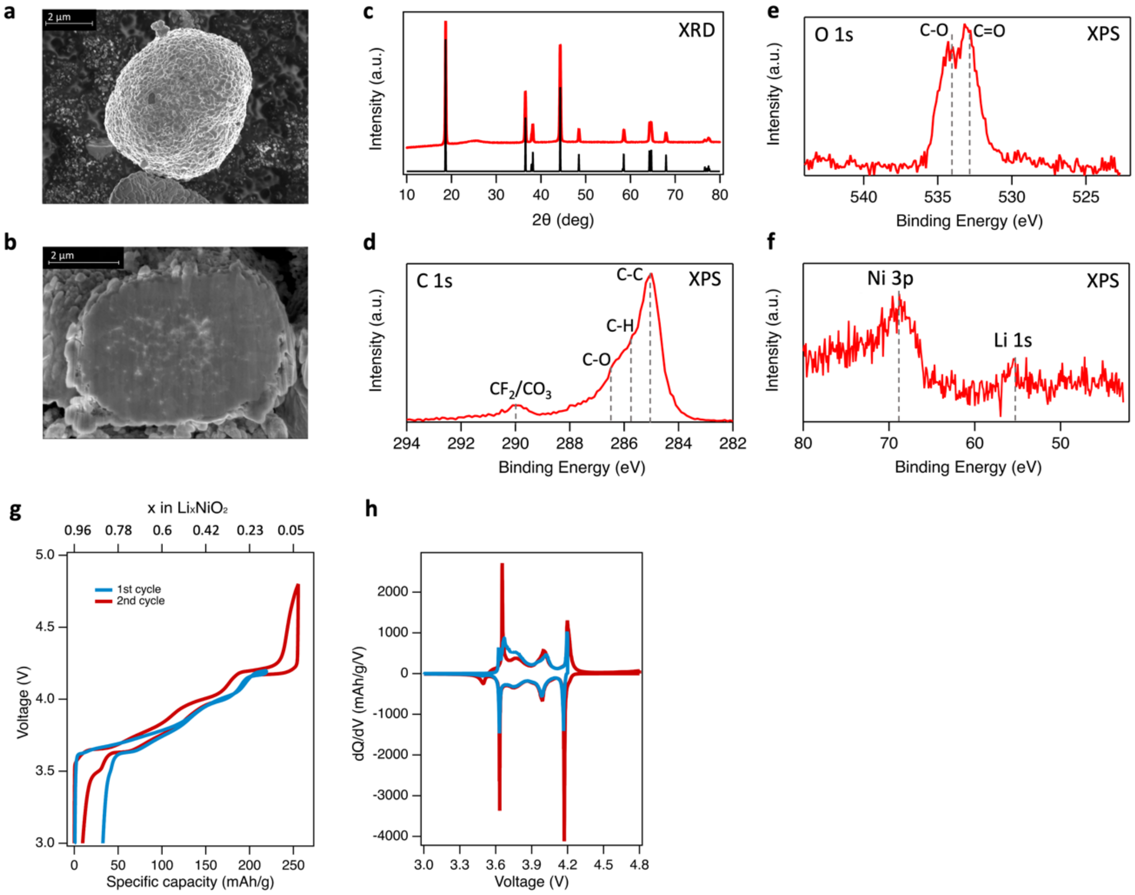

Supplementary Figure 1. Pristine $LiNiO_2$ characterisation. (a) SEM image of a pristine $LiNiO_2$ particle and (b) its cross section. (c) Lab-based X-ray powder diffraction pattern of pristine $LiNiO_2$ cathode (red), and theoretical powder diffraction pattern of $LiNiO_2$ with a rhombohedral structure (black).[N1] The additional broad peak in the measured diffraction pattern at ~26° corresponds to the acetylene black used as conductive agent in the cathode. (d) C 1s, (e) O 1s, (f) Ni 3p and Li 1s core-level spectra measured by lab-based XPS of the pristine $LiNiO_2$ cathode indicate the presence of Li_2CO_3 at the $LiNiO_2$ surface. (g) Typical first and second charge-discharge profiles of $LiNiO_2$ electrode cycled at a rate of C/20 vs. Li/Li^+ and (h) the corresponding differential capacity plots (dQ/dV).

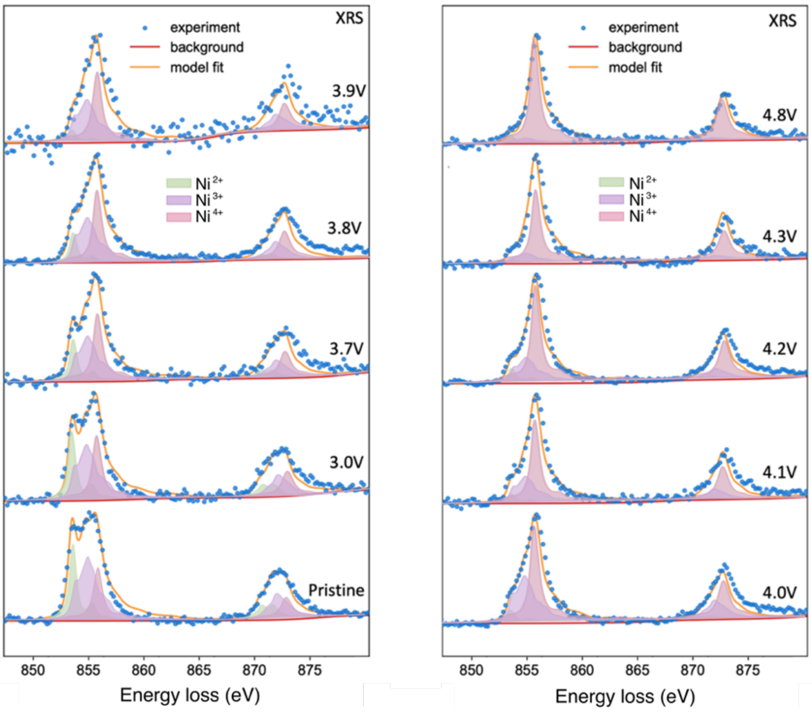

Supplementary Figure 2. Fitting results[N2] of experimental XRS Ni-$L_{3,2}$ edges using CTM calculated Ni^{2+}, Ni^{3+} and Ni^{4+} spectra.

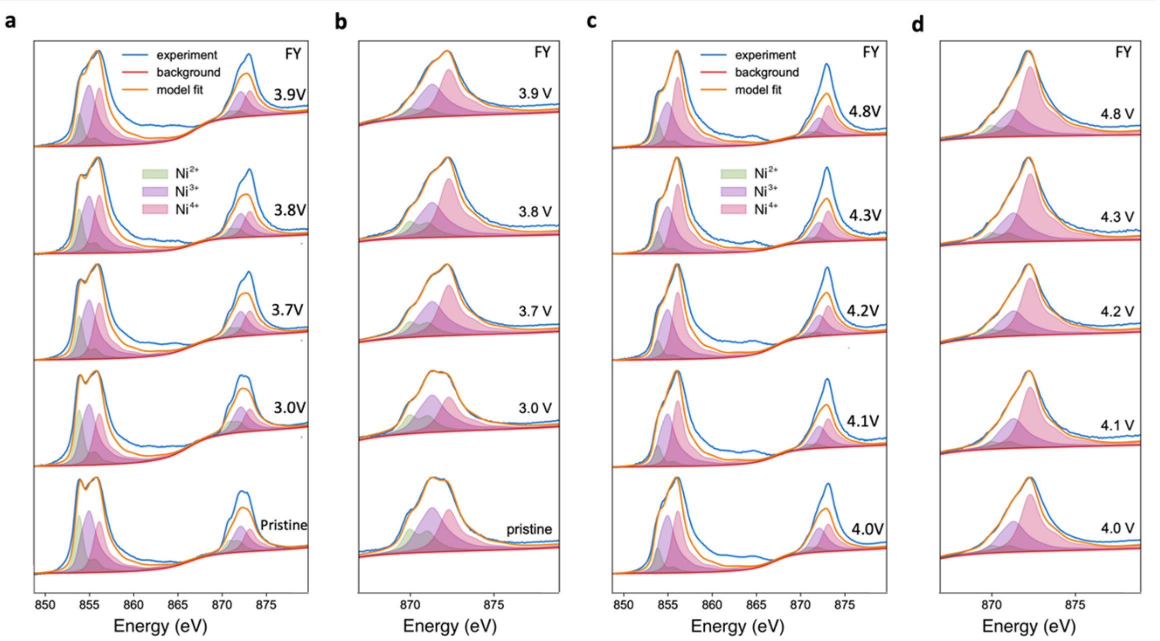

Supplementary Figure 3. Fitting results of experimental FY-XAS Ni-$L_{3,2}$ edges using CTM calculated Ni^{2+}, Ni^{3+} and Ni^{4+} spectra. Due to saturation and state-dependent decay effects in FY-XAS, the measured Ni-$L_{3,2}$ spectral shape is somewhat distorted.[1,2] Therefore, FY-XAS Ni-L_3 (a,c) and Ni-L_2 (b,d) edges are fitted separately using different broadening parameters (0.3 eV for L_3, 0.15 eV for L_2) and scaling.

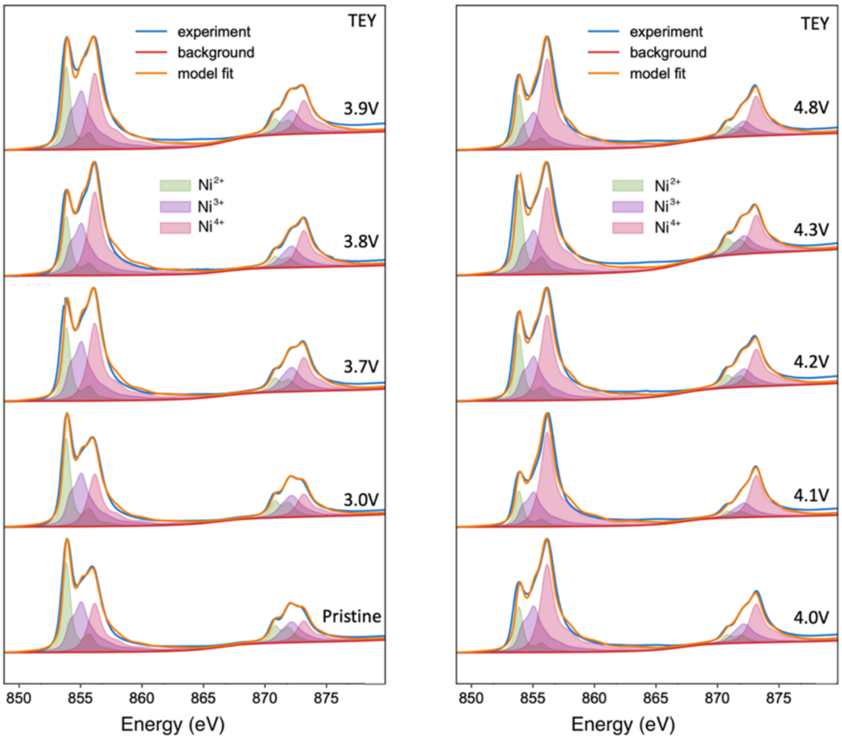

Supplementary Figure 4. Fitting results of experimental TEY-XAS Ni-$L_{3,2}$ edges using CTM calculated Ni^{2+}, Ni^{3+} and Ni^{4+} spectra.

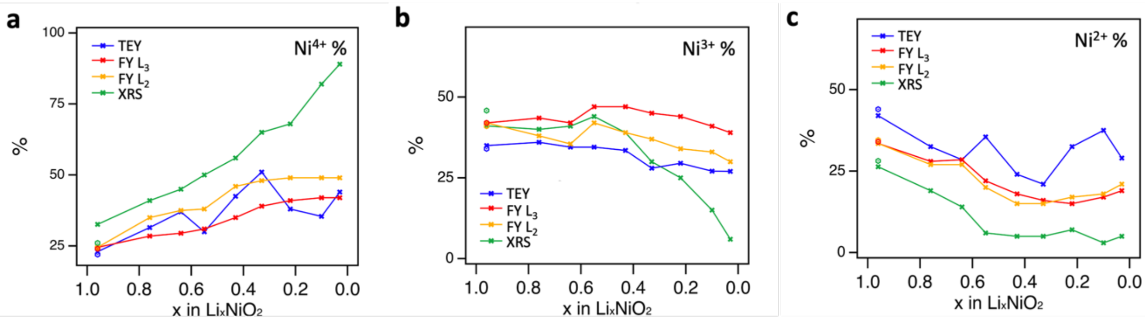

Supplementary Figure 5. Fitted percentages of (a) Ni^{4+}, (b) Ni^{3+} and (c) Ni^{2+} from Ni-L edges for $LiNiO_2$ at different states of charge (SoC). Values for pristine $LiNiO_2$ are indicated by hexagons to distinguish from those cycled to 3.0 V.

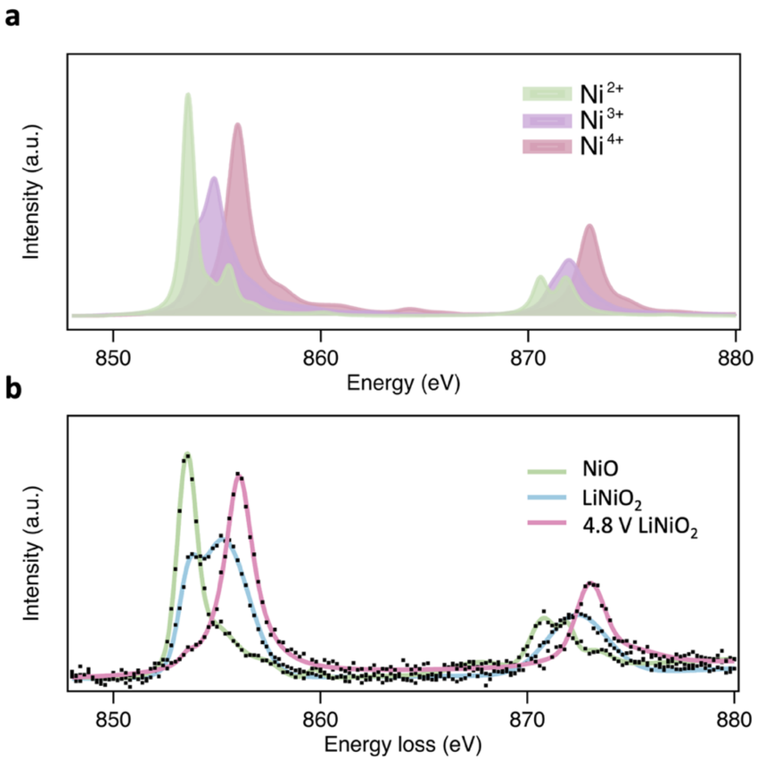

Supplementary Figure 6. (a) Calculated Ni $L_{3,2}$-edges of Ni^{2+}, Ni^{3+} and Ni^{4+}, and (b) XRS Ni $L_{3,2}$-edges of NiO, pristine $LiNiO_2$ and $LiNiO_2$ after charging to 4.8V vs. Li/Li^+. Each simulated spectrum is aligned to the peak feature of the corresponding experimental data.[N3] The pristine $LiNiO_2$ spectrum does not exclusively correspond to the Ni^{3+} species, as discussed in the main manuscript.

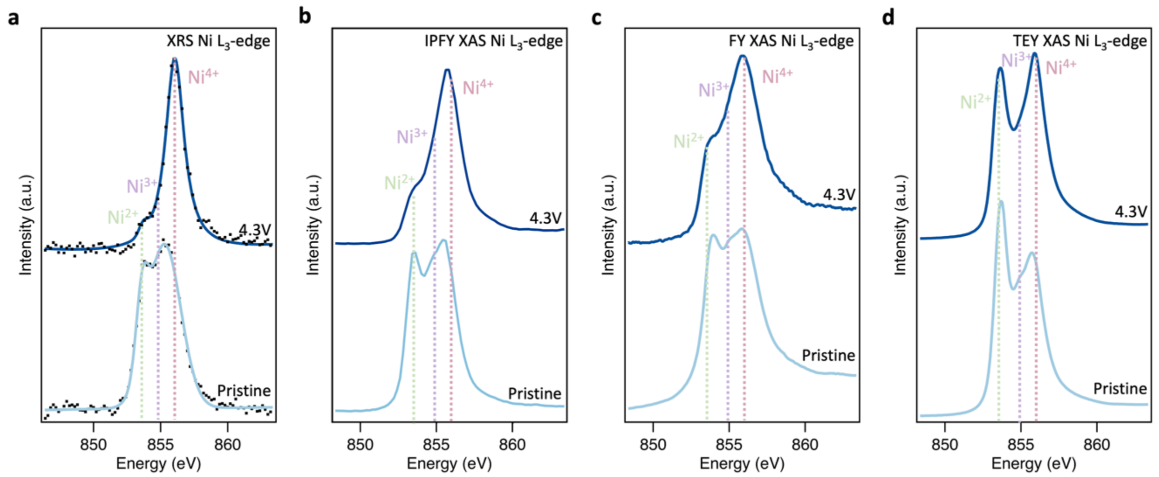

Supplementary Figure 7. Ni L_3-edges of pristine $LiNiO_2$ and after charging to 4.3 V vs. Li/Li^+ measured with (a) XRS, (b) IPFY-XAS, (c) FY-XAS, and (d) TEY-XAS. The differences in measured peak shapes between IPFY and FY are attributable to saturation and state-dependent decay effects,[1] which are less significant with IPFY.[2] Although this will influence the absolute values obtained from fitting FY-XAS, the observed trends with SoC for each fitted species should nevertheless persist.[3]

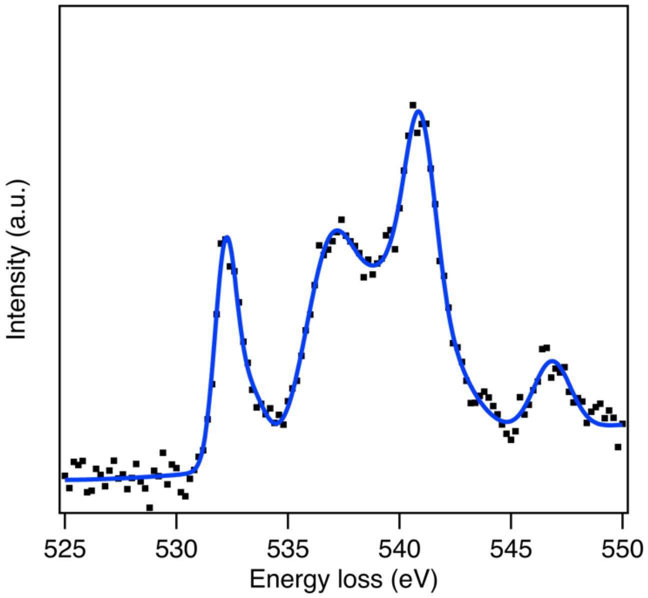

Supplementary Figure 8. XRS O K-edge of NiO. Experimental data is marked in dots with a smooth trace line.

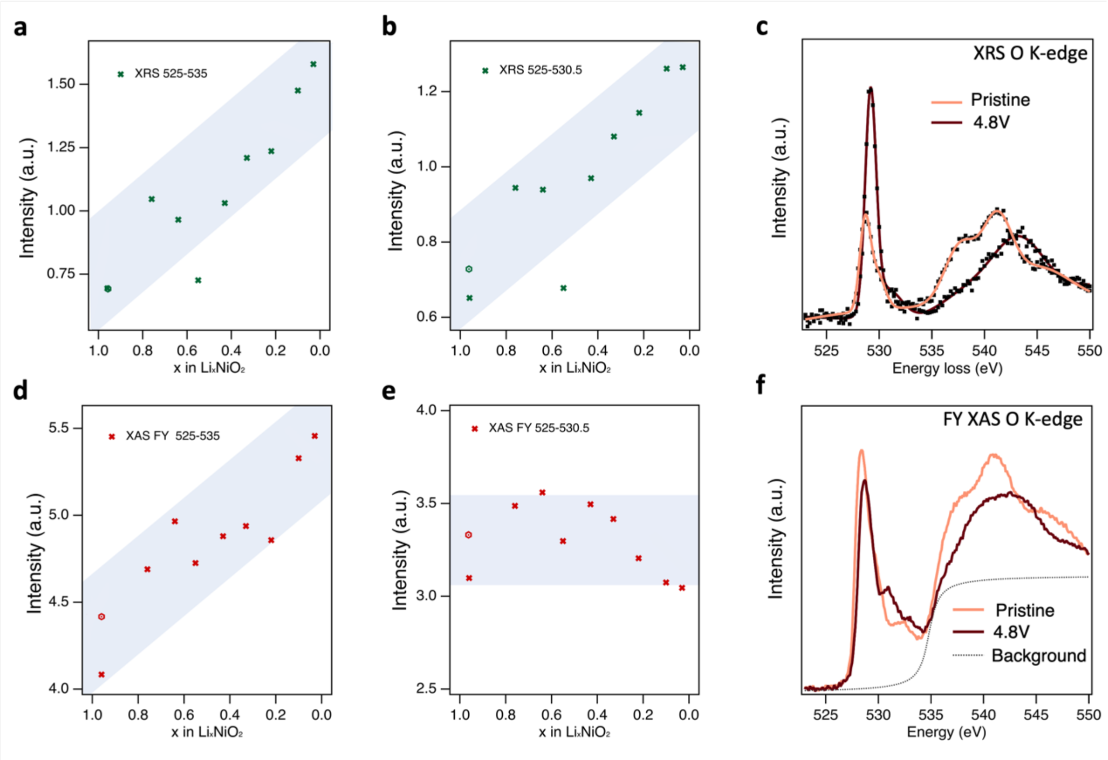

Supplementary Figure 9. Integrated pre-edge region of O K-edges from XRS (a, b) and FY-XAS (d-e) with different integration energy ranges.[N4] Values for pristine $LiNiO_2$ are indicated by hexagons to distinguish from those cycled to 3.0 V. The total area of the O K pre-edge region (525-535eV) from XRS and FY-XAS, and the area of just the first peak in the O K pre-edge region (525-530.5eV) from XRS all show increasing trends on delithiation. However, the first peak in the O K pre-edge region (525-530.5eV) from FY-XAS shows no such increase. O K-edge spectra of pristine $LiNiO_2$ and after charging to 4.8 V vs. Li/Li^+ from XRS (c) and FY-XAS (f).

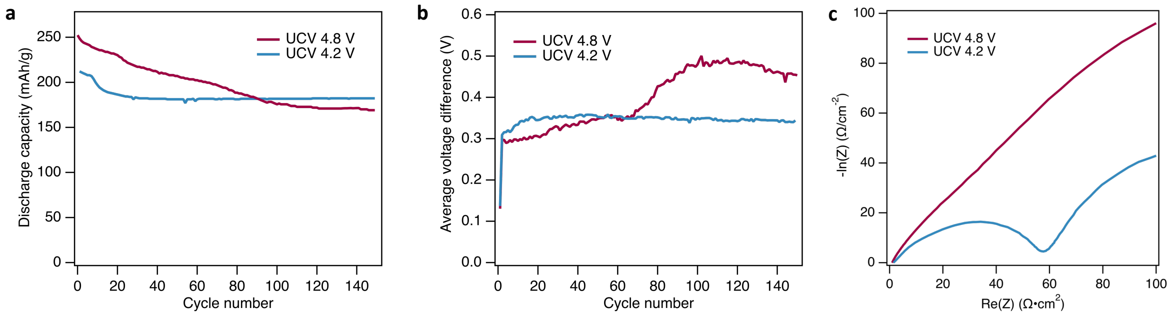

Supplementary Figure 10. Electrochemical performance of $LiNiO_2$ vs. Li cells cycled to upper cutoff voltages (UCV) of 4.8 V and 4.2 V showing (a) discharge capacity vs. cycle number, (b) voltage hysteresis between charge and discharge vs. cycle number. (c) Nyquist plots obtained from potential-controlled electrochemical impedance spectroscopy (EIS) after 150 cycles, performed at 3.8 V, with a 5 mV voltage perturbation in the frequency range of 1 MHz to 100 mHz. Each cell underwent an initial formation cycle [charge to 4.2 V at C/20, hold for 30 mins, and then discharge to 3.0 V at C/20], and were then cycled at C/5 for 150 cycles between UCV and 3.0 V. For EIS the cells were then cycled at C/20 to 3.8 V and held for 30 hours. Impedance values are normalised to the geometric area of the cathode.

Although the UCV of 4.8 V gives a higher initial discharge capacity compared to the UCV of 4.2V, the rate of capacity fade across 150 cycles is more rapid. There is a significant growth in the voltage hysteresis with cycling for a UCV of 4.8V compared to 4.2V, as well as EIS indicating a significantly higher charge transfer impedance, consistent with more extensive reduced surface layer (RSL) formation.

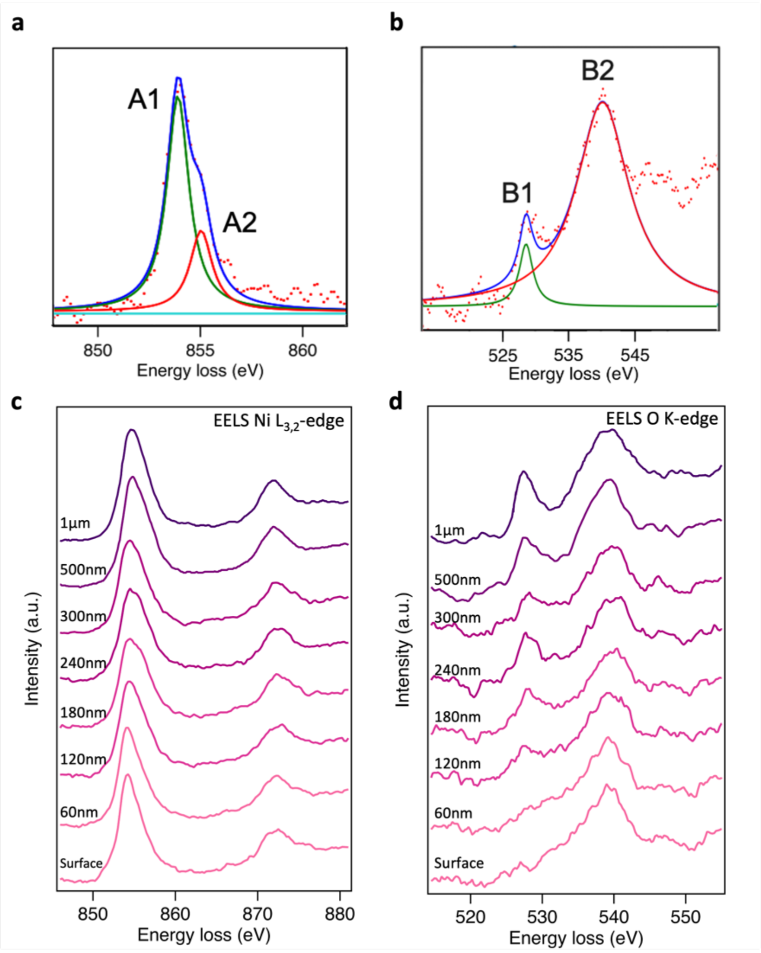

Supplementary Figure 11. Example peak fitting of (a) Ni L_3-edge and (b) O K-edge collected from the cross-sectional STEM-EELS. Hartree-Slater cross-sections are used for Ni L_3 edges with the continuum jump above the region shown in (a). (c) Ni $L_{3,2}$-edge and (d) O K-edge spectra acquired at different depths from the surface of a 4.8 V $LiNiO_2$ particle with STEM-EELS.

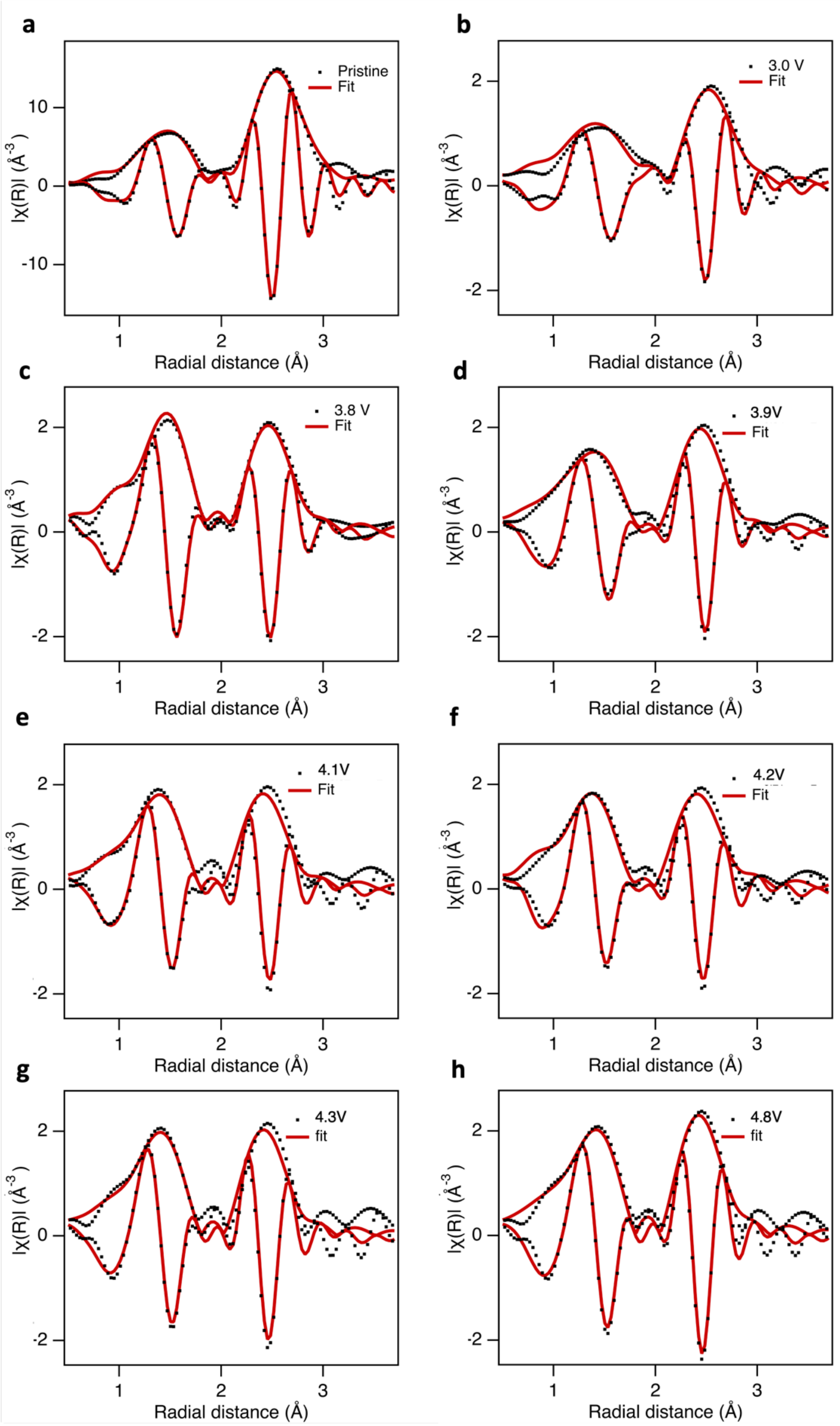

Supplementary Figure 12. (a-h) The amplitude and imaginary part fittings of k^2 weighted Fourier Transform-EXAFS spectra for $LiNiO_2$ at various SoC. A fitting range of 1-3.2 Å in R is used for all fits.[N5]

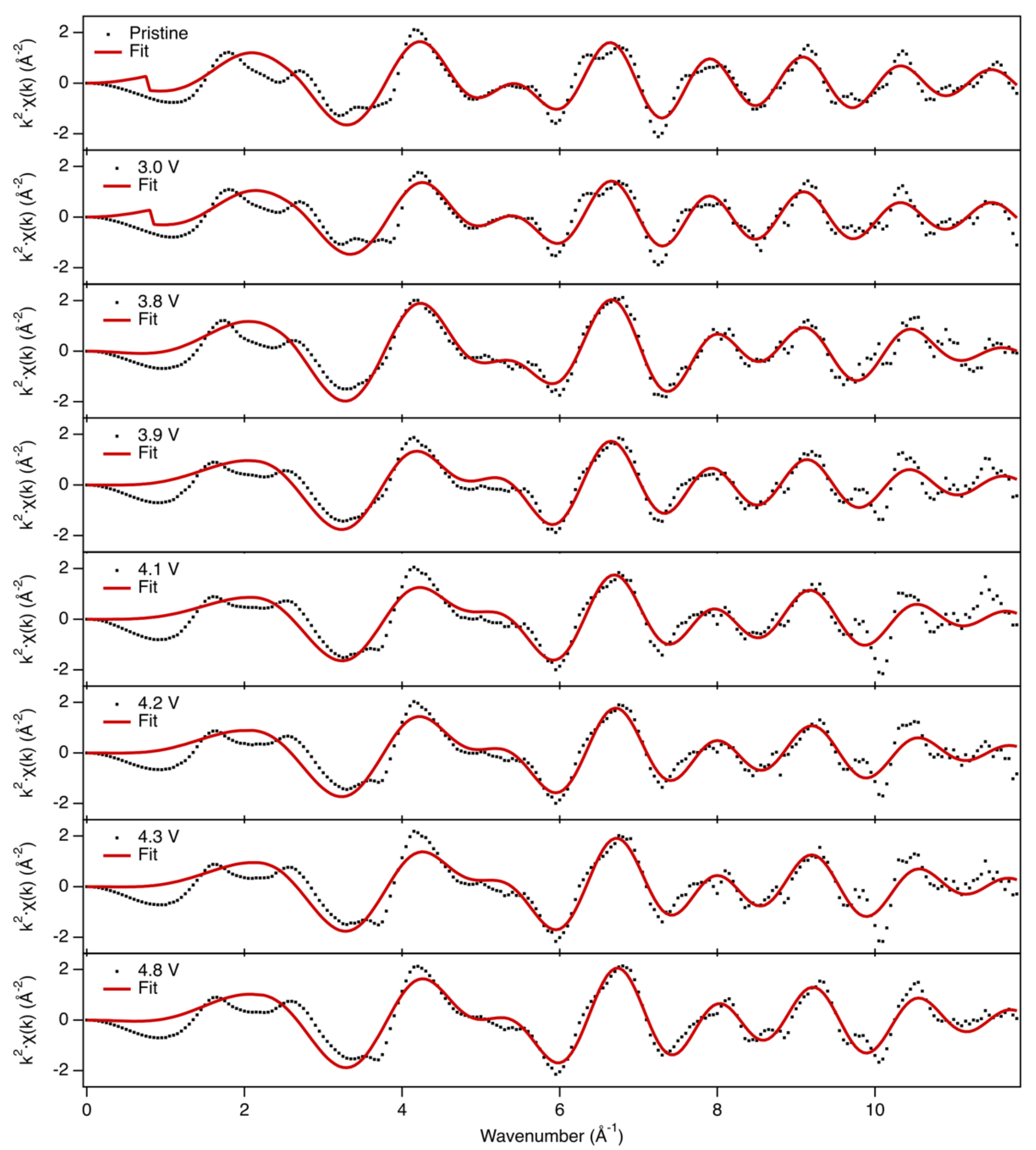

Supplementary Figure 13. EXAFS fittings for $LiNiO_2$ at various SoC, plotted as χ(k) in k space. A fitting range of 3.5-10.8 $Å^{-1}$ in k is used for all fits.

Supplementary Table 1. Fitted EXAFS structural parameters for $LiNiO_2$ at various SoC, corresponding to Supplementary Fig. 12,13, and used for Fig. 5f in the main text. Fitting of pristine, 3.0 V and 3.8 V $LiNiO_2$ adopted the disproportionated model which has short (S) and long (L) Ni-O bonds. The rest were each fitted with a single Ni-O distance.

Sample	Scattering path	R (Å)	σ^2 (Å^2)	E_0 (eV)	R factor
Pristine	Ni-O S	1.887	0.0023	2.27	0.17
	Ni-O L	2.052	0.0023		
	Ni-Ni	2.882	0.0043		
3.0 V	Ni-O S	1.869	0.0021	2.5	0.22
	Ni-O L	2.042	0.0021		
	Ni-Ni	2.885	0.0039		
3.8 V	Ni-O S	1.888	0.00158	-0.36	0.103
	Ni-O L	2.006	0.00158		
	Ni-Ni	2.850	0.00533		
3.9 V	Ni-O	1.866	0.008	-5.0	0.17
	Ni-Ni	2.835	0.006		
4.1 V	Ni-O	1.844	0.004	-6.1	0.21
	Ni-Ni	2.817	0.005		
4.2 V	Ni-O	1.846	0.005	-6.3	0.15
	Ni-Ni	2.813	0.005		
4.3 V	Ni-O	1.846	0.004	-4.9	0.19
	Ni-Ni	2.814	0.004		
4.8 V	Ni-O	1.856	0.004	-4.1	0.12
	Ni-Ni	2.816	0.004		

Supplementary Table 2. Comparison of EXAFS structural parameters obtained from fitting different models to pristine, 3.0 V and 3.8 V $LiNiO_2$. The J-T model maintains a fixed 2:1 ratio of short (S) to long (L) bonds. The disproportionated model used a fixed ratio of S to L bonds based on the fitting of CTM calculated spectra to XRS (main text Fig. 2). The floating model allowed the ratio of S to L bonds to freely vary. Coordination number is fixed to 6 for all fittings.

Sample/Model	Scattering path	R (Å)	σ^2 (Å^2)	Ni-O S:L ratio	E_0 (eV)	R factor
Pristine (single)	Ni-O	1.943	0.0119	N/A	1.5	0.172
	Ni-Ni	2.882	0.0052			
Pristine (J-T)	Ni-O S	1.904	0.0054	67:33 (fixed)	2.1	0.179
	Ni-O L	2.064	0.0054			
	Ni-Ni	2.885	0.0049			
Pristine (disproportionated)	Ni-O S	1.887	0.0023	58:42 (fixed)	2.3	0.166
	Ni-O L	2.052	0.0023			
	Ni-Ni	2.886	0.0043			
Pristine (floating)	Ni-O S	1.874	0.00009	52:48	2.2	0.164
	Ni-O L	2.042	0.00009			
	Ni-Ni	2.884	0.0038			
3.0 V (single)	Ni-O	1.929	0.0130	N/A	1.8	0.223
	Ni-Ni	2.882	0.0049			
3.0 V (disproportionated)	Ni-O S	1.869	0.0021	58:42 (fixed)	2.5	0.218
	Ni-O L	2.042	0.0021			
	Ni-Ni	2.885	0.0039			

3.0 V (floating)	Ni-O S	1.860	0.0007	53:47	2.5	0.219
	Ni-O L	2.032	0.0007			
	Ni-Ni	2.885	0.0039			
3.8 V (single)	Ni-O	1.91	0.005	N/A	-0.8	0.094
	Ni-Ni	2.85	0.005			
3.8 V (disproportionated)	Ni-O S	1.888	0.00158	75:25 (fixed)	-0.36	0.103
	Ni-O L	2.006	0.00158			
	Ni-Ni	2.850	0.00533			

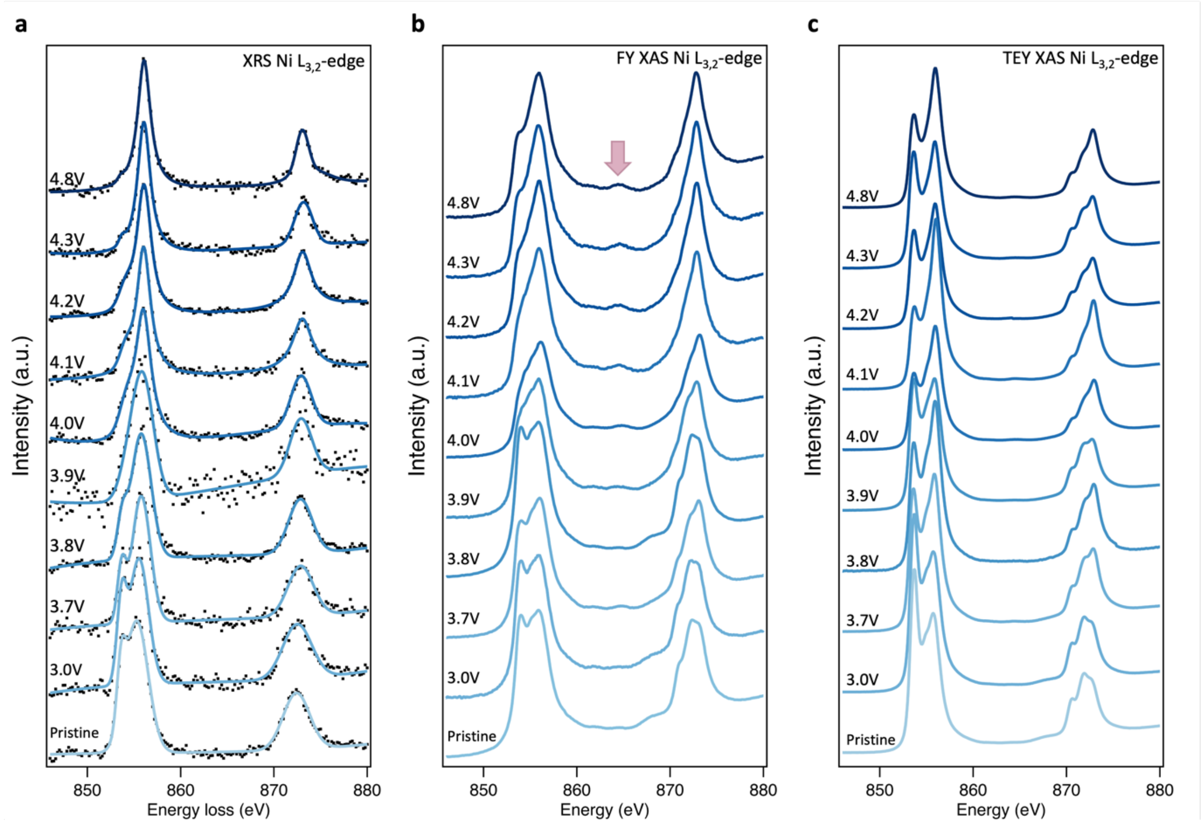

Supplementary Figure 14. Full Ni-$L_{3,2}$ edge spectra of $LiNiO_2$ at different SoC measured with (a) XRS, (b) FY-XAS and (c) TEY-XAS. The satellite features between Ni-L_3 and Ni-L_2 edge are indicated by an arrow in (b), with their greater relative intensity than in XRS or TEY attributable to state-dependent decay effects. The feature is present at all SoC, including the pristine $LiNiO_2$, and is most closely matched by the CTM calculated Ni^{4+} spectrum (see Supplementary Fig. 6), consistent with disproportionation.

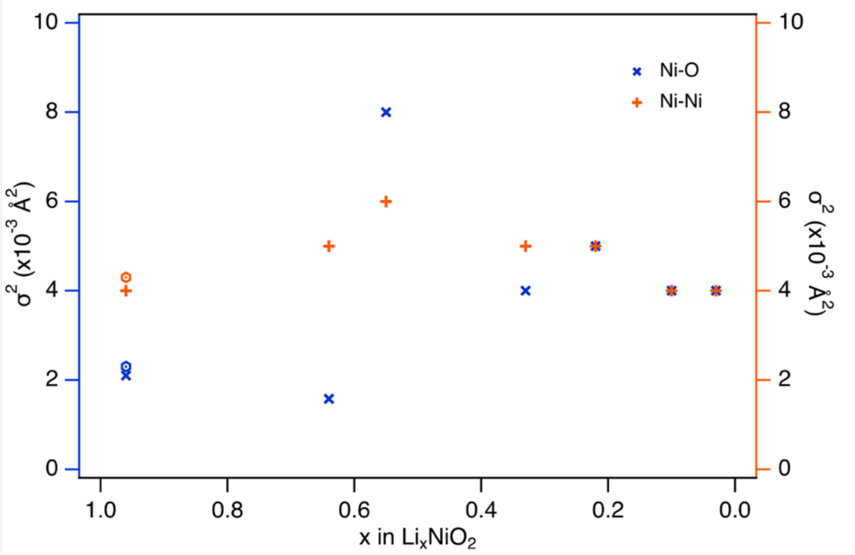

Supplementary Figure 15. Debye-Waller factors for fitting Ni-Ni and Ni-O distances of $LiNiO_2$ at different SoC from EXAFS data. Values for pristine $LiNiO_2$ are indicated by hexagons to distinguish from those cycled to 3.0 V vs. Li/Li^+. Ni-O distance fitting of pristine, 3.0 V and 3.8 V $LiNiO_2$ adopted the disproportionated model which has short and long Ni-O bonds. The rest were each fitted with a single Ni-O distance.

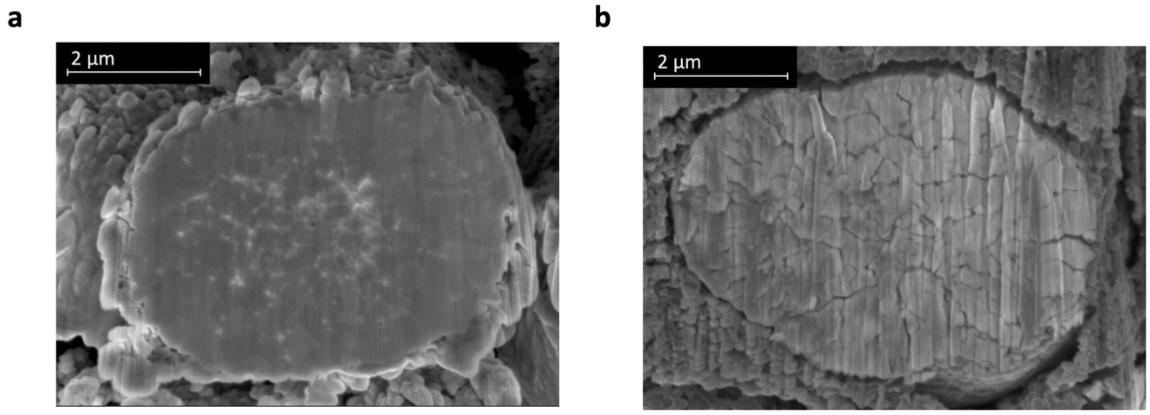

Supplementary Figure 16. Cross-sectional SEM images comparing (a) a pristine $LiNiO_2$ particle and (b) a $LiNiO_2$ particle cycled to 4.8V vs. Li/Li^+ that shows cracking between primary particles.

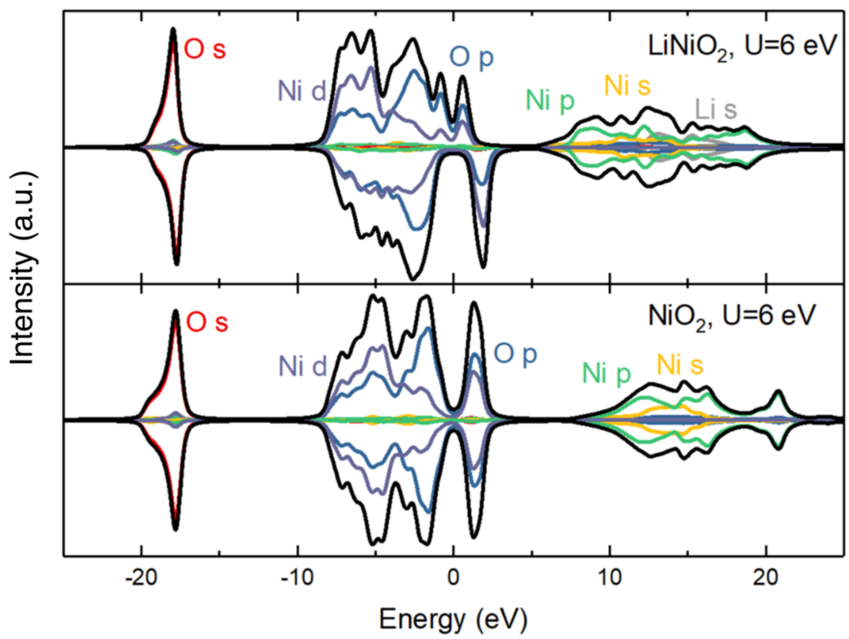

Supplementary Figure 17. Ground-state partial and total (black) density of states for $LiNiO_2$ (top) and NiO_2 (bottom) calculated using PBE+U (U=6 eV).[N6] Fermi energies are set to zero.

Supplementary Notes

N1. The structural parameters of pristine $LiNiO_2$ remain an area of debate, with several candidate models describing some of the experimentally observed properties. Whilst matching the rhombohedral ($R\overline{3}m$)[4] space group in room-temperature X-ray diffraction, more local probes such as EXAFS[5] and cryogenic-temperature neutron PDF[6] suggest local distortions of the NiO_6 octahedra in $LiNiO_2$. Such distortions are expected given the need to relieve orbital degeneracy in the formally d^7 spin-half Ni^{3+} species; disproportionation is an alternative way in which this could be achieved.[7,8] Complex models including random orientations of Jahn-Teller (J-T) orbital ordering have been considered, the static trimer structure (Pm) being one of the more popular to describe the experimentally observed PDF and electron structure factors,[6,9] although this is usually discounted on energetic grounds. Several lower energy structures incorporating J-T distortions of the NiO_6 have also been proposed as the ground state of $LiNiO_2$ including collinear monoclinic J-T (C2/m), noncollinear zig-zag J-T ($P2_1/c$, used here) which displays the lowest energy, and two-fold charge disproportionation (P2/c).[10–12] However, to reconcile the spatially averaged ($R\overline{3}m$) structure observed in techniques like XRD at room temperature with the local distortions seen in techniques like EXAFS, recent studies have suggested dynamically reorienting J-T effects,[13,14] and dynamic three-fold disproportionation,[7] all of which allow for local J-T distortions whilst preserving an undistorted structure on average. The energy difference between many of the minimum energy static structures considered above (C2/m, $P2_1/c$, P2/c and disproportionation) are far below 30 meV, indicating at least that at room temperature some dynamic structural reorientation may be possible. For the modelling of core-level spectra in this study (Fig. 5), we have opted for the lowest energy $P2_1/c$ structure, noting that structures with similarly non-equivalent bond lengths are expected to generate broadly similar spectra.

N2. To fit the XRS, FY-XAS, and TEY-XAS (Supplementary Fig. 2-4), an arctan background with L_3-L_2 separation fixed at 17.3 eV centred near the continuum edge jump in pristine $LiNiO_2$ was used to represent the transitions to the continuum. The strength of the background and contributions of three Ni species were allowed to change to accommodate the saturation of the FY L_3 edge. The spectral shapes from CTM calculations were broadened by 0.1 eV for XRS and TEY fitting, by 0.3 eV for FY L_3, and 0.15 eV for FY L_2. Due to the multitude of free parameters, the fitting is approximate. Relative species concentrations are estimated within about 2%, which is a deviation at which the fits are visibly inaccurate.

N3. Multiplet calculations were performed in Quanty[15] using the single-cluster Hamiltonian described by Green et al. [15] taking the form:

$$H_{LF} = H_U^{dd} + H_U^{pd} + H_{l\cdot s}^{d} + H_{l\cdot s}^{p} + H_{OS}^{d} + H_{OS}^{p} + H_{OS}^{L} + H_{hyb}^{dL} H_{LF}$$

$$= H_U^{dd} + H_U^{pd} + H_{l\cdot s}^{d} + H_{l\cdot s}^{p} + H_{OS}^{d} + H_{OS}^{p} + H_{OS}^{L} + H_{hyb}^{dL}$$

where H_U represents the atomic Coulomb repulsion between either dd or pd electrons including multiplet effects, $H_{l\cdot s}$ the spin-orbit interaction of the 3d or 2p orbitals, H_{OS} the onsite energy of the 2p, 3d and ligand orbitals, and H_{hyb}^{dL} the hybridisation or interaction energy between d and L orbitals. Implementation of the onsite energies utilises the site symmetry of the Ni atom, which was chosen to be O_h (symmetric) in the nominally Ni^{2+} ($n_d = 8$) and Ni^{4+} ($n_d = 6$) case, and D_{4h} (representing a splitting due to the Jahn-Teller effect) for the Ni^{3+} ($n_d = 7$). Here, all three systems are treated as having the same monopole Coulomb interaction parameters ($U_{dd} = 6$ eV and $U_{dd} = 7$ eV) and the same Slater integrals and spin-orbit parameters (matching the Ni^{3+} configuration determined from atomic Hartree-Fock calculations,[16] which is to effectively assume that the three species form a non-interacting multi-cluster environment of the same material (no mixing is accounted for). Slater integrals are scaled to 80% and 85% for the initial and final Hamiltonians respectively. Additionally,

onsite ligand energy shifts of T_{pp}=±0.75 eV were applied to the ligand orbitals of e_g (+) and t_{2g} (-) symmetry for all cluster configurations.

For Ni^{2+}, parameterization of the CTM ligand field model is well established for NiO.[17] We used these as the basis for our calculations but employed slightly larger hopping integrals and crystal field splitting to account for the comparatively shorter bond lengths in $LiNiO_2$. Other parameters were allowed to vary by a few percent relative to the established model to achieve better fits to the data. Parameters used in the Ni^{2+} calculation (eV) were Δ= 5.5, $10D_q$= 0.71, V_{eg}= 2.627, V_{t2g}= 1.524. For Ni^{3+}, parameter values for the perovskite nickelates were used as a starting value,[15] and modified slightly to account for the Jahn-Teller distortion. Ni^{3+} has also been compared with sodium nickelate[18] where disproportionation is not expected. Parameters used in the Ni^{3+} calculation (eV) were Δ = -0.5, $10D_q$= 0.93 with Jahn-Teller splitting of Δ_{eg}= 0.15 and Δ_{t2g}= 0.10 where Δ_{eg} is the difference between the $x^2 - y^2$ and $3x^2 - r^2$ onsite energies and Δ_{t2g} is the difference between the xy and xz/yz onsite energies, $V_{3z^2-r^2}$= 2.43, $V_{x^2-y^2}$= 3.33, $V_{xz/yz}$= 1.41, V_{xz}= 1.93. For Ni^{4+}, hopping integrals and crystal field energies were employed as an extrapolation of those from Ni^{2+} and Ni^{3+} to match experimental data for Ni^{4+}. Parameters used in the Ni^{4+} calculation (eV) were Δ= -6.5, $10D_q$= 0.78, V_{eg}= 3.456, V_{t2g}=2.004.

N4. Before integration, FY-XAS O K-edges are first normalised and then subtracted with an arctan background as displayed in Supplementary Fig. 9f. We note that the XRS signal to noise ratio is lower than that of the FY-XAS, particularly for x=0.54 due to sample variation. The increasing trends of integrated pre-edge area thus shows some scatter. Nevertheless, they clearly reveal that the growth in total O K pre-edge area seen with XRS is the result of growth in the first pre-edge peak area (525-530.5 eV), which is associated with increased Ni-O covalency (i.e. lower electron density on O sites) as $LiNiO_2$ is delithiated. Conversely, the same comparison for the FY-XAS confirms that the growth in total O K pre-edge area instead primarily relates to the emergence of the

molecular O_2 feature (530.5-535 eV) that is associated with near-surface degradation.

N5. EXAFS fitting was carried out using the Artemis software of the Demeter package in the original EXAFS signal (k) space. Scattering paths were calculated from crystal structure data of $LiNiO_2$ ($R\bar{3}m$) using FEFF code v6L. The coordination numbers (N) for both N-O and N-Ni single scattering paths were fixed to their crystal structure values (6 and 6 respectively). A fitting range of 1-3.2 Å in R is used for all fits. Changes in absorber-backscatterer distance (ΔR) and mean-square displacements, often referred to as the Debye-Waller factor (σ^2) were refined for each scattering path while the amplitude reduction factor (S_0^2) and the energy shift (ΔE_0) were refined globally.

N6. For the DFT calculations in this work, we chose not to implement any on-site coulomb interaction correction (i.e. Hubbard U) despite a typical value of $U \approx 6$ eV being used for $LiNiO_2$ in the literature.[11,19] Despite the Hubbard U treatment being successful in capturing electronic properties such as the band gap in nickelate systems, this was found to artificially distort O K pre-edge spectra due to the redistribution of d density (O 2p has strong d orbital hybridization) giving a poorer match to the experimental data relative to the pure PBE functional. Using hybrid or meta-GGA functionals could improve upon this at greater computational expense.

Supplementary References

1. de Groot, F. M. F., Arrio, M. A., Sainctavit, P., Cartier, C. & Chen, C. T. Fluorescence yield detection: Why it does not measure the X-ray absorption cross section. *Solid State Commun* **92**, 991–995 (1994).
2. Asakura, D. *et al.* Material/element-dependent fluorescence-yield modes on soft X-ray absorption spectroscopy of cathode materials for Li-ion batteries. *AIP Adv* **6**, (2016).
3. Achkar, A. J. *et al.* Bulk sensitive x-ray absorption spectroscopy free of self-absorption effects. *Phys Rev B Condens Matter Mater Phys* **83**, 2–5 (2011).
4. Rougier, A., Gravereau, P. & Delmas, C. Optimization of the Composition of the Li1 − z Ni1 + z O 2 Electrode Materials: Structural, Magnetic, and Electrochemical Studies. *J Electrochem Soc* **143**, 1168–1175 (1996).
5. Rougier, A., Delmas, C. & Chadwick, A. V. Non-cooperative Jahn-Teller effect in LiNiO2: An EXAFS study. *Solid State Commun* **94**, 123–127 (1995).
6. Chung, J.-H. *et al.* Local structure of LiNiO2 studied by neutron diffraction. *Phys Rev B* **71**, 064410 (2005).
7. Poletayev, A. D., Cottom, J. P., Morgan, B. J. & Islam, M. S. Temperature-Dependent Dynamic Disproportionation in LiNiO2. (2022).
8. Foyevtsova, K., Elfimov, I., Rottler, J. & Sawatzky, G. A. LiNiO2 as a high-entropy charge- and bond-disproportionated glass. *Phys Rev B* **100**, 165104 (2019).
9. Cao, J., Zou, H., Guo, C., Chen, Z. & Pu, S. Local trimer orbital ordering in LiNiO2 studied by quantitative convergent beam electron diffraction technique. *Solid State Ion* **180**, 1209–1214 (2009).
10. Radin, M. D. & Van Der Ven, A. Simulating Charge, Spin, and Orbital Ordering: Application to Jahn-Teller Distortions in Layered Transition-Metal Oxides. *Chemistry of Materials* **30**, 607–618 (2018).
11. Chen, H., Freeman, C. L. & Harding, J. H. Charge disproportionation and Jahn-Teller distortion in LiNiO2 and NaNiO2: A density functional theory study. *Phys Rev B Condens Matter Mater Phys* **84**, 85108 (2011).
12. Chen, Z., Zou, H., Zhu, X., Zou, J. & Cao, J. First-principle investigation of Jahn-Teller distortion and topological analysis of chemical bonds in LiNiO2. *J Solid State Chem* **184**, 1784–1790 (2011).
13. Sicolo, S., Mock, M., Bianchini, M. & Albe, K. And Yet It Moves: LiNiO2, a Dynamic Jahn-Teller System. *Chemistry of Materials* **32**, 10096–10103 (2020).
14. Genreith-Schriever, A. R. *et al.* Jahn-Teller Distortions and Phase Transitions in LiNiO2: Insights from Ab Initio Molecular Dynamics and Variable-Temperature X-ray Diffraction. *Chemistry of Materials* **36**, 2289–2303 (2023).
15. Green, R. J., Haverkort, M. W. & Sawatzky, G. A. Bond disproportionation and dynamical charge fluctuations in the perovskite rare-earth nickelates. *Phys Rev B* **94**, 195127 (2016).
16. Haverkort, M. W. Spin and orbital degrees of freedom in transition metal oxides and oxide thin films studied by soft x-ray absorption spectroscopy. (2005).
17. Haverkort, M. W., Zwierzycki, M. & Andersen, O. K. Multiplet ligand-field theory using Wannier orbitals. *Phys Rev B Condens Matter Mater Phys* **85**, 165113 (2012).
18. Green, R. J. *et al.* Evidence for bond-disproportionation in LiNiO2 from x-ray absorption spectroscopy. (2020).
19. Genreith-Schriever, A. R. *et al.* Oxygen Hole Formation Controls Stability in LiNiO2 Cathodes. *Joule* **7**, 1623–1640 (2023).
